# Reducing histone acetylation rescues cognitive deficits in a mouse model of Fragile X syndrome

**DOI:** 10.1038/s41467-018-04869-3

**Published:** 2018-06-27

**Authors:** Yue Li, Michael E. Stockton, Brian E. Eisinger, Yinghua Zhao, Jessica L. Miller, Ismat Bhuiyan, Yu Gao, Zhiping Wu, Junmin Peng, Xinyu Zhao

**Affiliations:** 1 Waisman Center, University of Wisconsin-Madison, Madison, WI 53705 USA; 2 Department of Neuroscience, University of Wisconsin-Madison, Madison, WI 53705 USA; 3 Departments of Structural Biology and Developmental Neurobiology, St. Jude Proteomics Facility, St. Jude Children’s Research Hospital, 262 Danny Thomas Place, Memphis, TN 38105 USA; 4 Present Address: Institute of Traditional Chinese Medicine Research, Tianjin University of Traditional Chinese Medicine, Tianjin, 300193 China

## Abstract

Fragile X syndrome (FXS) is the most prevalent inherited intellectual disability, resulting from a loss of fragile X mental retardation protein (FMRP). Patients with FXS suffer lifelong cognitive disabilities, but the function of FMRP in the adult brain and the mechanism underlying age-related cognitive decline in FXS is not fully understood. Here, we report that a loss of FMRP results in increased protein synthesis of histone acetyltransferase EP300 and ubiquitination-mediated degradation of histone deacetylase HDAC1 in adult hippocampal neural stem cells (NSCs). Consequently, FMRP-deficient NSCs exhibit elevated histone acetylation and age-related NSC depletion, leading to cognitive impairment in mature adult mice. Reducing histone acetylation rescues both neurogenesis and cognitive deficits in mature adult FMRP-deficient mice. Our work reveals a role for FMRP and histone acetylation in cognition and presents a potential novel therapeutic strategy for treating adult FXS patients.

## Abstract (web summary)

Loss of fragile X mental retardation protein (FMRP) leads to fragile X syndrome, associated with cognitive dysfunction. Here the authors show that mice lacking FMRP show reduced hippocampal neurogenesis and cognitive deficits, which can be rescued by reducing histone acetylation.

## Introduction

Fragile X syndrome (FXS), resulting from CGG expansion in the fragile X mental retardation (*FMR1*) gene and functional loss of fragile X mental retardation protein (FMRP), is the most prevalent single gene cause of autism and heritable developmental disability, affecting 1 in 4000 males and 1 in 6000 females worldwide^1^. FMRP is a brain-enriched RNA-binding protein and plays a critical role in learning and memory^2–6^. Extensive efforts have been devoted to the understanding of the function of FMRP in neurons, which led to a number of therapeutic targets and clinical trials^6–8^. However, the outcomes have been disappointing, and no Food and Drug Administration (FDA) approved treatment is available^9,10^. More importantly, although FXS is categorized as a neurodevelopmental disorder, FXS patients have a normal lifespan, and their neurological symptoms and cognitive deficits persist throughout their adult life into old age. However, nearly all FXS research has focused on developing and young adult brains. The role of FMRP in cognitive maintenance in mature and older adults remains unexplored. Therefore, identifying novel mechanisms underlying FMRP regulation of brain function, especially those relevant to mature adult brain functions, is important for both a complete understanding of FMRP function in the brain and for developing effective treatments.

Extensive evidence from animal and human studies support potentially important roles of hippocampal neurogenesis for information processing, acquisition of new memories, and behavioral adaptations to the ever-changing environment^11^. Neurogenesis is altered in a number of neurodevelopmental and neuropsychiatric disorders, including aging^11–16^. Therefore, strategies targeting adult neurogenesis have been proposed as potential therapies for treating human brain disorders with cognitive dysfunctions; however, such treatment remains absent. Hippocampal neurogenesis requires a permanent pool of neural stem cells (NSCs) that exist in a balanced state of quiescence versus activation^15,17,18^. Abnormally elevated NSC activation leads to exhaustion of the NSC pool over time^19,20^. It has been shown that hippocampal neurogenesis in the rodent declines with age, and much of this decline is a result of reduction in the number of NSCs^11^. Recently, age-dependent decline in adult new neuron production has also been shown in humans^21^. Therefore, restoring the NSC pool might be a sound strategy for enhancing neurogenesis and cognitive functions in older adults with cognitive deficits. We have shown that FMRP deficiency leads to aberrant adult NSC activation and cognitive deficits in young adult mice^14^; however its impact on the adult NSC pool and cognitive maintenance in mature adult brains remains unexplored.

Acetylation of histone H3 and H4, controlled by balancing the activity of histone acetyltransferases (HATs) and histone deacetylases (HDACs), is one of the key epigenetic modifications controlling gene expression^22^. Both HATs and HDACs are critical for brain development^23^ and altered histone acetylation has been found in embryonic cortical neurons lacking FMRP^24^. In adult brains, histone acetylation also has important roles in brain plasticity and cognitive functions, and abnormal histone acetylation profiles are associated with psychiatric and neurological disorders, including aging^25^. However, whether abnormal histone modifications contribute to cognitive deficits seen in FXS patients remains unknown.

Here, we showed that a loss of FMRP led to depletion of hippocampal NSCs and age-related cognitive impairment. We showed that the loss of FMRP resulted in elevated protein synthesis of histone acetyltransferase EP300 and downregulation of histone deacetylase HDAC1 through ubiquitin-mediated degradation. Both elevated EP300 and reduced HDAC1 resulted in abnormally high levels of histone H3 and H4 acetylation. Rebalancing histone acetylation by either reducing EP300 activities or enhancing HDAC1 levels rescued both neurogenic and cognitive deficits in fragile X syndrome mice. Our data reveal a previously unknown role for FMRP and balanced histone acetylation in maintaining the NSC pool and adult cognitive ability and present new therapeutic targets for treating FXS.

## Results

### FMRP deficiency leads to the depletion of NSCs in mature adults

**Fig. 1:**
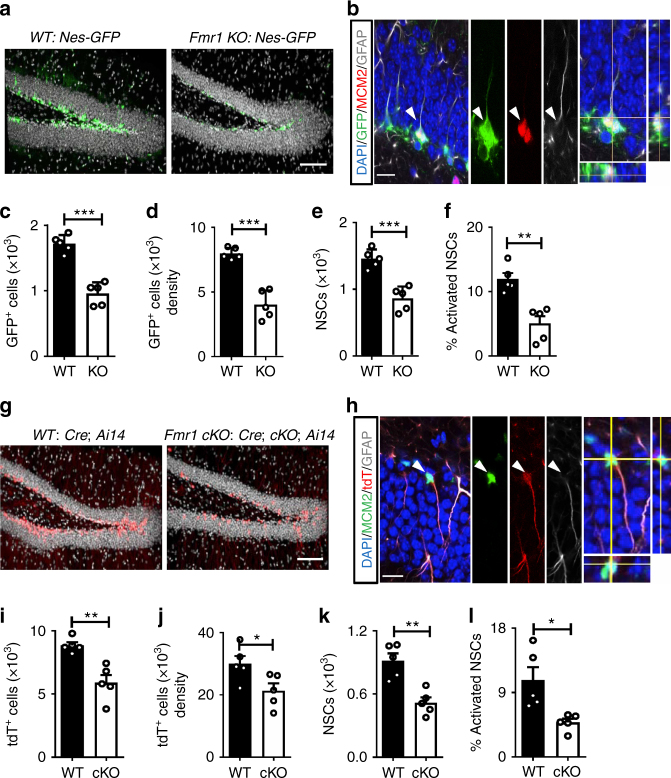
FMRP deficiency leads to hippocampal NSC depletion in mature adult mice. **a** Sample confocal images of brain sections from 6-month-old *WT;Nes-GFP* and *Fmr1 KO;Nes-GFP* mice. Gray, DAPI (4’,6-diamidine-2-phenylindole dihydrochloride); green, GFP. Scale bar, 100 µm. **b** Sample confocal images used for identifying NSCs (GFP^+^GFAP^+^) and activated NSCs (GFP^+^GFAP^+^MCM2^+^) in the DG of adult *Fmr1* KO and WT mice bred onto a *Nes-GFP* background. Green, GFP; red, MCM2; white, GFAP. Scale bar, 20 µm. White arrowheads, activated NSCs. **c**, **d** Quantitative comparison of the number (**c**) and the density (**d**, number mm^−3^) of GFP^+^ cells in the DG of 6-month-old adult *Fmr1* KO mice and WT littermate controls. **e**, **f** Quantitative comparisons of the number of NSCs (**e**) and percentage of activated NSCs (**f**) in the DG of *Fmr1* KO mice and WT littermate controls. **g** Sample confocal images of brain sections from *cKO;Cre;Ai14* mice and *Cre;Ai14* control mice. Gray, DAPI; red, tdTomato (tdT) Scale bar, 100 µm. **h** Sample confocal images used for identifying NSCs (tdT^+^GFAP^+^) and activated NSCs (tdT^+^GFAP^+^MCM2^+^) in the DG of *cKO;Cre;Ai14* mice and *Cre;Ai14* control mice. Red, tdT; green, MCM2; white, GFAP. Scale bar, 20 µm. **i**, **j** Quantitative comparison of the number (**i**) and the density (**j**, number mm^−3^) of tdT^+^ cells in the DG of 6-month-old adult *cKO;Cre;Ai14* mice and *Cre;Ai14* control mice. **k**, **l** Quantitative comparisons of the number of NSCs (**k**) and percentage of activated NSCs (**l**) in the DG of *cKO;Cre;Ai14* mice and *Cre;Ai14* control mice. *P < 0.05, **P < 0.01, ***P < 0.001. Student’s *t*-tests were used for data analyses. Data are presented as mean ± s.e.m.

We have previously found that 2-month-old (young adult) *Fmr1* knockout (KO) mice have increased NSC activation in the dentate gyrus (DG) of the hippocampus, which is coupled to reduced neuronal differentiation, overall reduction in neurogenesis, and cognitive impairment^14^. Since high levels of NSC activation may lead to NSC depletion in the adult brain^19,20^; we investigated whether FMRP is important in maintaining the NSC pool in adult mice. Because adult hippocampal neurogenesis declines from 2 to 6 months of age in mice, which is associated with reduction of cognitive abilities^26,27^, we decided to assess NSC maintenance in 6-month-old (6-mo) mature adult mice. We used *Fmr1* mutant (KO) mice crossed with *Nestin* promoter-driven green fluorescent protein (*Nes*-GFP) transgenic mice in which GFP was expressed in both NSCs and intermediate progenitors (IPCs) (Fig. 1a, b). We found that 6-mo *Fmr1* KO mice had a decreased number (Fig. 1c) and density (Fig. 1d) of GFP^+^ cells in the DG compared to their wild-type (WT) littermates, without significant changes in the overall volume of the DG (Supplementary Fig. 1a). We next analyzed different populations of GFP^+^ cells in the subgranular zone. *Fmr1* KO mice had 40.8% fewer type 1 radial glia-like NSCs (GFP^+^GFAP^+^) (Fig. 1b, e) and 63.9% fewer IPCs (TBR2+ or GFP+ GFAP−) (Supplementary Fig. 1c–e) compared to their WT littermates. Using MCM2 as a marker for cell cycle initiation (Fig. 1b, Supplementary Fig. 1b), we found that the proportion of activated NSCs (GFP^+^GFAP^+^MCM2^+^) was significantly lower in *Fmr1* KO mice compared with age-matched WT mice (Fig. 1f). *Fmr1* KO also had reduced proliferation (Ki67^+^) in both NSCs and IPCs when compared to WT mice (Supplementary Fig. 1f–h).

To determine whether NSC depletion was due to FMRP intrinsic regulation of NSCs, we crossed *Fmr1*-floxed (*cKO*) mice with inducible *Nestin* promoter (*Nes*)-driven Cre transgenic mice (*Nes-CreER*^*T2*^) and *Rosa26-STOP-tdTomato* (*Ai14*) reporter mice to create triple transgenic mice (*Nes-CreER*^*T2*^*;cKO;Ai14*, simplified as *Cre;cKO;Ai14*) and control littermates (*Cre;Ai14*) (Supplementary Fig. 2a). Tamoxifen injections of 2-month-old mice leads to targeted deletion of FMRP and selective expression of tdTomato (tdT) specifically in adult NSCs and their subsequent progenies^14^ (Fig. 1g, h). We found that at 4 months post tamoxifen injection, 6-month-old *Cre;cKO;Ai14* mice had significantly fewer number (Fig. 1i) and lower density (Fig. 1j) of tdT+ cells, fewer NSCs (Fig. 1k), and lower proportion of activated NSCs (Fig. 1l) compared to control *Cre;Ai14* mice without significant changes in the overall volume of the DG (Supplementary Fig. 2b). Thus, in contrast to our previous finding that FMRP deficiency led to increased NSC activation in 2-month-old (young adult) FMRP-deficient mice^14^, a loss of FMRP leads to depletion of NSCs and decreased NSC activation when mice reach 6 months of age (mid-age). These results suggest that FMRP plays a critical role in the maintenance of NSCs in the adult hippocampus.

### FMRP controls EP300 protein synthesis

**Fig. 2:**
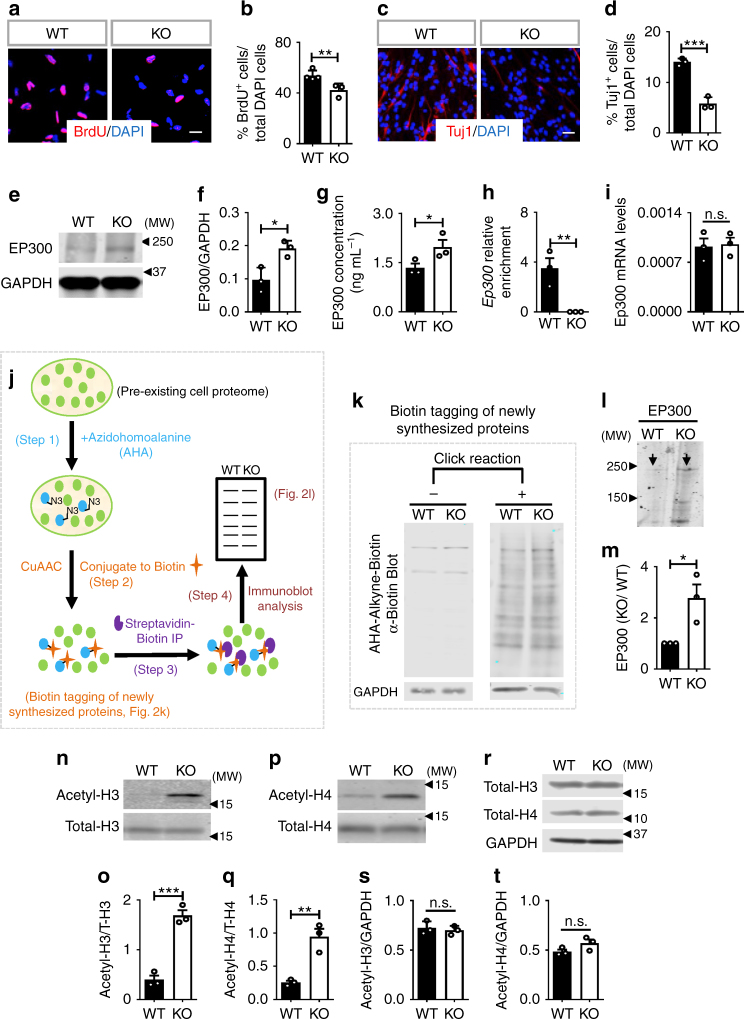
FMRP controls EP300 protein synthesis in NPCs of 6-month old mice. **a**, **b** *Fmr1 KO* 6-mo NPCs exhibited reduced proliferation compared to WT NPCs as assessed by incorporation of the thymidine analog, BrdU (**a**, red, BrdU; blue, DAPI. scale bar, 20 µm) and quantitative analysis (**b**, *n* = 3–4). **c**, **d** *Fmr1 KO* 6-mo NPCs differentiated into fewer neurons compared to WT NPCs as assessed by a neuronal marker Tuj1^+^ (**c**, red, BrdU; blue, DAPI. scale bar, 20 µm) followed by quantitative analysis (**d**, *n* *=* 3). **e**, **f** Western blot analyses of EP300 protein expression levels in WT and *Fmr1* KO 6-mo NPCs, (*n* = 3). GAPDH was used as a loading control. **g** ELISA analyses of EP300 protein levels in WT and *Fmr1* KO 6-mo NPCs (*n* = 3). **h** RNA-IP-qPCR analysis showing FMRP was associated with *Ep300* mRNA in NPCs (*n* *=* 3). **i** Quantitative real-time PCR analyses showing no significant differences in *Ep300* mRNA levels between WT and *Fmr1* KO 6-mo NPCs (*n* = 3). **j** Workflow for detection and identification of newly synthesized proteins using BONCAT (bio-orthogonal non-canonical amino acid tagging). **k** Visualization of Biotin-tagged, newly synthesized proteins in WT and *Fmr1* KO NPCs using Biotin blot. **l**, **m** Sample western blot (upper panel) and quantitative analysis (lower panel, *n* = 3) of newly synthesized EP300 protein in WT and *Fmr1* KO NPCs as determined by using BONCAT. **n**–**q** Western blot analyses of acetylation of histone H3 (**n**, **o**) and H4 (**p**, **q**) in WT and *Fmr1* KO 6-mo NPCs (*n* *=* 3). Total histone H3 or H4 was used as a loading control for acetylation of histones. **r**–**t** Western blot analyses of total histone H3 and H4 levels in WT and *Fmr1* KO NPCs (*n* = 3). GAPDH was used as a loading control. *P < 0.05, **P < 0.01, ***P < 0.001. n.s.: no significant difference. Student’s *t*-tests were used for data analyses. Data are presented as mean ± s.e.m.

To explore the molecular mechanism underlying NSC depletion in FMRP-deficient mice, we assessed NPCs isolated from the DG of 6-mo *Fmr1* KO and littermate control mice (Supplementary Fig. 3a) and found that 6-mo *Fmr1* KO NPCs exhibited decreased proliferation and decreased neuronal differentiation compared with WT NPCs (Fig. 2a–d), which is consistent with our in vivo observations (Fig. 1). We then used these 6-mo NPCs to investigate the mechanism underlying FMRP regulation of NSC maintenance in mature adult mice. Among the predicted FMRP targets in dividing cells^2^, MDM2 and EP300 are ranked as the top 1 and 2 regulators for NSC activation and maintenance^14^. We found that although the levels of both MDM2 and activated MDM2 (phosphorylated- or P-MDM2) were elevated in 6-mo *Fmr1* KO NPCs compared to WT cells (Supplementary Fig. 3b–d), the levels of the canonical target of MDM2, P53, exhibited no significant difference (Supplementary Fig. 3e, f). On the other hand, EP300 protein levels were significantly elevated in 6-mo (Fig. 2e–g) Fmr1 KO NPCs. The elevated EP300 was not found in either hippocampal and cortical tissues dissected from 6-mo *Fmr1* KO mice (Supplementary Fig. 3g, h) or in NPCs isolated from the DG of 2-mo *Fmr1* KO mice (Supplementary Fig. 3i, j). We therefore first investigated whether upregulation of EP300 contributes to NPC impairment specific to 6-mo NPCs.

To determine whether FMRP directly regulates EP300, we performed RNA-binding protein immunoprecipitation and identification of co-precipitated RNA (RNA-IP) with an FMRP antibody coupled with real-time PCR analysis. We found that FMRP associated with messenger RNAs (mRNAs) of *Ep300* (Fig. 2h), as well as a positive control *Gsk3β*, but not a negative control *Gapdh* in NPCs (Supplementary Fig. 4a, d). Since *Ep300* mRNA levels were not altered (Fig. 2i) and FMRP is known to bind its mRNA target and regulate protein translation^5,6^, we next determined whether FMRP regulated EP300 protein synthesis in 6-mo NPCs. We employed a bio-orthogonal non-canonical amino acid tagging (BONCAT) method allowing for specific detection of newly synthesized proteins in cells through biotin tagging (Fig. 2j)^28,29^. We found that *Fmr1* KO NPCs had more newly synthesized EP300 protein compared with WT NPCs (Fig. 2k–m), suggesting increased EP300 protein synthesis. We also detected increased protein synthesis of glycogen synthase kinase-3β (GSK3β), a known translational target of FMRP, in adult NPCs^13^, but not for control GAPDH^14^ (Supplementary Fig. 5a, b, e, f). Therefore, our data support a mechanism in which FMRP binds *Ep300* mRNA and represses its protein synthesis in NPCs.

EP300 is a transcriptional co-activator with HAT activity. We found that in *Fmr1* KO NPCs, the levels of acetylated histone H3 and H4, but not total H3 or H4, were >2.5× fold higher compared to WT NPCs (Fig. 2n–t). Corroborating this finding, the mRNA levels of cyclin-dependent kinase (CDK) inhibitor *p16*, a known transcriptional target of EP300^30,31^ was also significantly higher in *Fmr1* KO compared to WT NPCs (Supplementary Fig. 6). Therefore, these results suggest that FMRP maintains the balance of histone acetylation in NSCs through translational regulation of EP300.

### FMRP maintains HDAC1 protein levels in NPCs

**Fig. 3:**
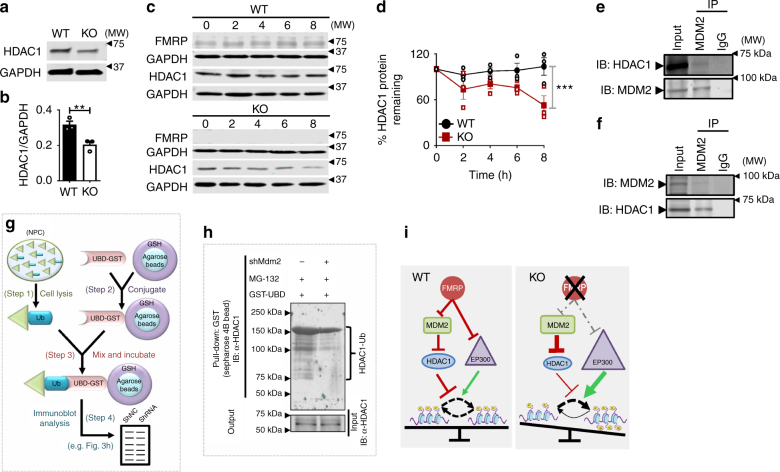
FMRP maintains HDAC1 protein levels in NPCs of 6-month-old mice. **a**, **b** Western blot analyses of HDAC1 protein levels in WT and *Fmr1* KO 6-mo NPCs (*n* = 3). **c**, **d** HDAC1 protein stability in WT and *Fmr1* KO NPCs treated with the protein synthesis inhibitor cycloheximide (CHX) over an 8 h period. Two-way ANOVA (*n* = 3, *F*(4, 20) = 17.86, ***P < 0.001; Time × genotype interaction, *F*(4, 20) = 2.53, *P* = 0.0727; Time, *F*(4, 20) = 2.006, *P* = 0.1324). Note that FMRP protein levels in WT cells did not change during the treatment. **e**, **f** Co-immunoprecipitation analysis showed that endogenous HDAC1 physically associated with endogenous MDM2 in 6-mo NPCs. **g** Workflow for detection of ubiquitination of HDAC1 using GST-labeled ubiquitin-binding domain (UBD) pull down. **h** GST-UBD pull down of NPCs with MDM2 knockdown (+shMdm2) and control (−shMdm2) followed by western blotting with a HDAC1 antibody showing that NPCs with reduced MDM2 levels also had reduced ubiquitination of HDAC1. Cells were treated with MG132 to inhibit proteasome degradation of ubiquitinated proteins. **i** Schematic models of FMRP balancing histone acetylation through regulation of MDM2-HDAC1 and EP300 pathways. **P < 0.01, ***P < 0.001, except for Fig. 3d, Student’s *t*-tests were used for data analyses. Data are presented as mean ± s.e.m.

We next investigated whether elevated MDM2 and P-MDM2 could also affect histone acetylation levels in 6-mo NPCs. Since the levels of P53, the canonical target of MDM2, did not change in 6-mo *Fmr1* KO NPCs (Supplementary Fig. 3), we searched for other potential candidate MDM2 targets that may regulate NSC activation and maintenance. We compared a list of 335 “Neurogenic Regulators” we have curated previously^14^ to a list of 31 proteins that are either downstream targets or cooperating proteins of MDM2 based on literature^32^. We identified a set of 17 common factors which represented candidates mediating MDM2 regulation of NSC activation. Candidates were ranked by the number of literature-supported interactions with neurogenic regulators, reflecting the likelihood of their involvement in NPC activity. Among them, EP300, NUMB, XIAP, CBP, and HDAC1 were the top 5, while P53 was ranked number 6 (Supplementary Data 1, Supplementary Fig. 7). We found that neither mRNA levels nor protein levels of NUMB, XIAP, and CBP exhibited differences in *Fmr1* KO NPCs. However, *Fmr1* KO NPCs had significantly reduced HDAC1 protein levels compared to WT NPCs (Fig. 3a, b and Supplementary Fig. 8).

Next, we determined whether FMRP directly regulates HDAC1. We found that FMRP was not associated with *Hdac1* mRNA but was associated with *Mdm2* mRNA (Supplementary Fig. 4b, c) in NPCs. Indeed, FMRP repressed MDM2 protein synthesis as assessed by BONCAT assay (Supplementary Fig. 5c, d). Since MDM2 catalyzes ubiquitination and degradation of HDAC1 in other cell types^33^ and HDAC1 protein levels were significantly reduced in *Fmr1* KO NPCs (Fig. 3a), we investigated whether HDAC1 downregulation in *Fmr1* KO NPCs was a result of elevated MDM2 levels. We first assessed protein stability of HDAC1 in NPCs in the presence of cycloheximide to block protein synthesis. Indeed, HDAC1 protein levels decreased significantly faster in *Fmr1* KO NPCs compared to WT NPCs during an 8 h culturing period (Fig. 3c, d). Next, using co-immunoprecipitation followed by western blotting analysis, we confirmed that endogenous MDM2 physically interacted with endogenous HDAC1 in NPCs (Fig. 3e, f). To determine whether MDM2 catalyzes ubiquitination of HDAC1 in NPCs, we acutely knocked down MDM2 using lentivirus small hairpin RNA (shRNA; s*hMdm2*) in WT NPCs and collected protein lysate in the presence of a proteasome inhibitor (MG132) to preserve ubiquitinated proteins. We then pulled down total ubiquitinated proteins from the same amount of WT or KO NPC protein lysate using the same amount of purified glutathione *S*-transferase (GST)-tagged four concatenated ubiquitin-binding domains (UBDs)^34^ and then assessed the levels of ubiquitinated HDAC1 in the pull-down samples using western blotting (Fig. 3g). We found that NPCs with MDM2 knockdown (*shMdm2*) had reduced ubiquitination of HDAC1 compared to NPCs treated with control (*shNC*) (Fig. 3h), while global ubiquitination remained the same (Supplementary Fig. 9). These results suggest that MDM2 mediates ubiquitination and degradation of HDAC1 in NPCs, and that elevated MDM2 is likely the reason why HDAC1 levels are downregulated in *Fmr1* KO NPCs. Therefore, these results indicate that FMRP maintains the balance of histone acetylation by regulating both EP300 and HDAC1 (Fig. 3i).

### Rebalancing histone acetylation rescues FMRP-deficient NPCs

**Fig. 4:**
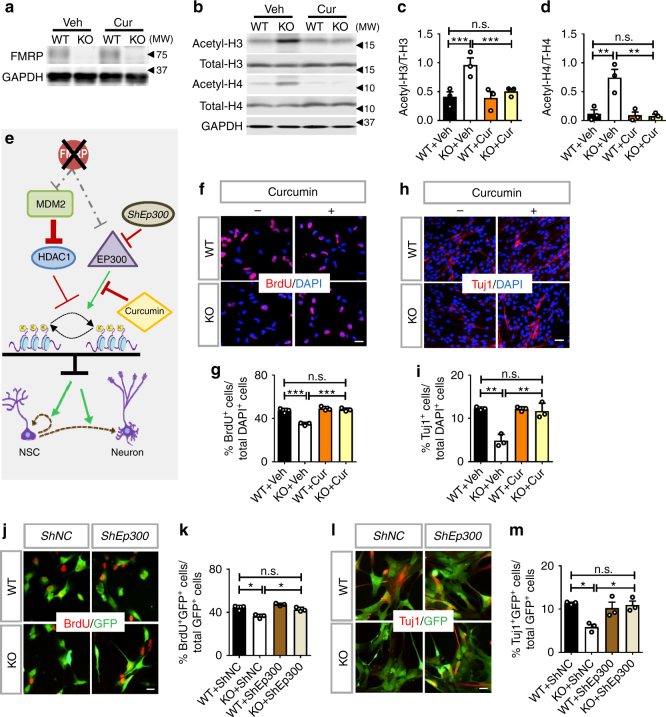
Reducing EP300 activities rebalances histone acetylation and rescues proliferation and differentiation deficits of FMRP-deficient NPCs. **a** FMRP protein expression in primary *Fmr1* KO and WT NPCs treated with vehicle or curcumin (Cur). **b** A schematic model showing that reducing EP300 activities either pharmacologically or genetically rebalances histone acetylation and rescues proliferation and differentiation of *Fmr1* KO NPCs. **c**–**e** Western blot analyses of total and acetylation of histone H3 (**c**, **d**) and histone H4 (**c**, **e**) in NPCs treated with curcumin (*n* *=* 3). Total histone H3 and total histone H4 were used as loading controls for acetylated H3 and H4, respectively. **f**, **g** Curcumin treatment rescued the cell proliferation phenotype of *Fmr1* KO NPCs as assessed by incorporation of the thymidine analog, BrdU (**f**, red, BrdU; blue, DAPI. scale bar, 20 µm) and quantitative analysis (**g**, *n* = 3). **h**, **i** Curcumin treatment rescued neuronal differentiation phenotypes of *Fmr1* KO NPCs as assessed by a neuronal marker Tuj1^+^ (**h**, red, Tuj1; blue, DAPI. scale bar, 20 µm) followed by quantitative analysis (**i**, *n* *=* 3). **j**, **k** Acute knockdown of EP300 rescued the cell proliferation phenotype of *Fmr1* KO NPCs as assessed by incorporation of the thymidine analog, BrdU (**j**, red, BrdU; green, GFP, scale bar, 20 µm) and quantitative analysis (**k**, *n* = 3). **l**, **m** Acute knockdown of EP300 rescued neuronal differentiation phenotypes of *Fmr1* KO NPCs as assessed by a neuronal marker Tuj1^+^ (**l**, red, Tuj1; green, GFP, scale bar, 20 µm) followed by quantitative analysis (**m**, *n* *=* 3). *P < 0.05, **P < 0.01, ***P < 0.001. n.s.: no significant difference. Two-way ANOVAs were used for data analyses. Data are presented as mean ± s.e.m.

We next determined whether rebalancing histone acetylation might correct deficits of *Fmr1* KO NPCs. We decided to inhibit EP300 activity using two known chemical inhibitors, curcumin^35^ and C646^36^. Curcumin, a natural product and food color, has been evaluated extensively for its therapeutic potential and mechanism of action in human cancers and neurodegenerative disease, and is currently in a number of clinical trials including Alzheimer’s disease^35,37^ (https://clinicaltrials.gov). C646 is a synthetic small molecule designed to inhibit EP300 with high specificity^36^. Based on literature^38^ and pilot experiments, we selected the dosages for curcumin or C646 that exhibit minimal or no effect on WT cells. We found that both curcumin (Fig. 4a–e) and C646 (Supplementary Fig. 10a–e) treatment reduced acetylation of histone H3 and H4 in *Fmr1* KO NPCs to levels similar to those in WT NPCs, with no significant effect on WT NPCs. We then determined whether they could rescue neurogenic deficits of *Fmr1* KO NPCs. Indeed, both curcumin and C646 treatment enhanced proliferation (Fig. 4f, g and Supplementary Fig. 10f, g) and neuronal differentiation (Fig. 4h, i and Supplementary Fig. 10h, i) of *Fmr1* KO NPCs without affecting WT NPCs. To further validate the results of pharmacological inhibition, we next used a small hairpin inhibitory RNA that could partially knock down EP300 (*shEp300*) (Supplementary Fig. 11) and found that genetic reduction of EP300 also rescued proliferation and differentiation deficits of *Fmr1* KO NPCs (Fig. 4j–m). Hence, reducing the activities of EP300 rebalances histone acetylation and rescues proliferation and differentiation deficits of *Fmr1* KO NSCs.

**Fig. 5:**
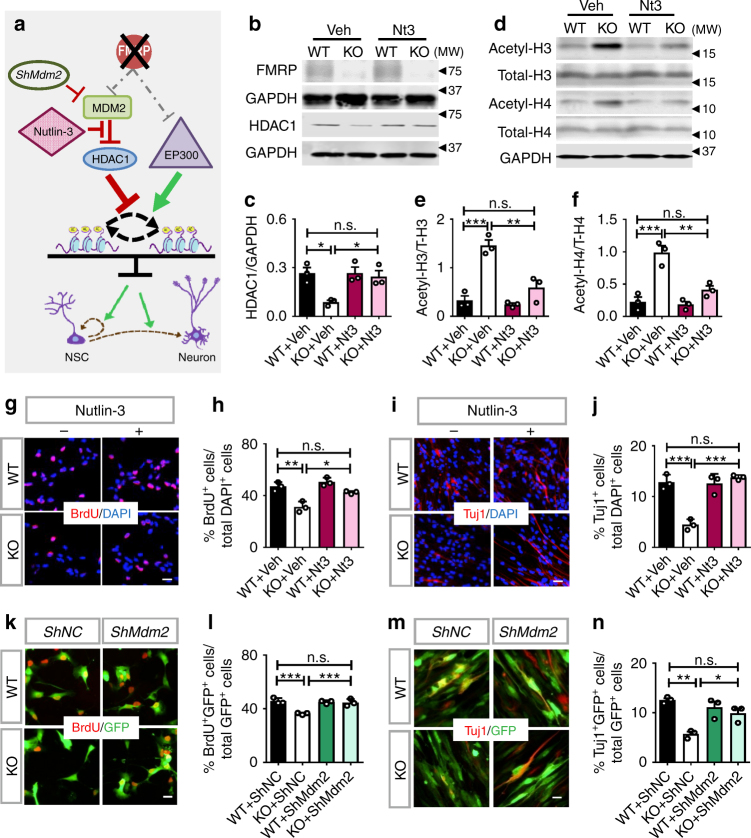
Increasing HDAC1 rebalances histone acetylation and rescues proliferation and differentiation deficits of FMRP-deficient NPCs. **a** A schematic model showing that enhancing HDAC1 activities through either pharmacological or genetic reduction of MDM2 rebalances histone acetylation and rescues proliferation and differentiation of *Fmr1* KO NPCs. **b**, **c** Western blot analyses of HDAC1 and FMRP protein levels in 6-mo NPCs treated with Nutlin-3 (*n* = 3). GAPDH was used as a loading control. **d**–**f** Western blot analyses of total and acetylated histone H3 (**d**, **e**) and histone H4 (**d**, **f**) in NPCs treated with Nutlin-3 (*n* *=* 3). Total histone H3 and total histone H4 were used as loading controls for acetylated H3 and H4, respectively. **g**, **h** Nutlin-3 treatment rescued the cell proliferation phenotype of *Fmr1* KO NPCs as measured by BrdU incorporation (**g**, red; scale bar, 20 µm) followed by quantitative analysis (**h**, *n* = 3). **i**, **j** Nutlin-3 treatment rescued neuronal differentiation phenotypes of *Fmr1* KO NPCs as assessed by Tuj1^+^ staining (**i**, red; scale bar, 20 µm) followed by quantitative analysis (**j**, *n* *=* 3). **k**, **l** Acute knockdown of MDM2 rescued the cell proliferation phenotype of *Fmr1* KO NPCs as assessed by incorporation of the thymidine analog, BrdU (**l**, red, BrdU; green, GFP, scale bar, 20 µm) and quantitative analysis (**l**, *n* = 3). **m**, **n** Acute knockdown of EP300 rescued neuronal differentiation phenotypes of *Fmr1* KO NPCs as assessed by a neuronal marker Tuj1^+^ (**m**, red, Tuj1; green, GFP, scale bar, 20 µm) followed by quantitative analysis (**n**, *n* *=* 3). *P < 0.05, **P < 0.01, ***P < 0.001, n.s.: no significant difference. Two-way ANOVA was used for all data analyses. Data are presented as mean ± s.e.m.

We then determined whether rebalancing histone acetylation through enhancing HDAC1 activity might correct deficits of *Fmr1* KO NPCs. We used Nutlin-3, a compound in a phase 1 clinical trial for the treatment of retinoblastoma^39^, to inhibit MDM2. Although Nutlin-3 is designed to inhibit MDM2–P53 interaction, it has been shown to inhibit MDM2 interaction with other substrates, including HDAC1^33,40^. Using a dosage of Nutlin-3 with minimal effect on WT cells^14^, we found that Nutlin-3 did not affect P53 levels in either *Fmr1* KO or WT cells (Supplementary Fig. 12), but increased HDAC1 levels in *Fmr1* KO cells with no significant effect on WT cells (Fig. 5a–c). In addition, Nutlin-3 treatment restored histone acetylation levels in *Fmr1* KO NPCs (Fig. 5d–f) and rescued proliferation and neuronal differentiation of *Fmr1* KO NPCs without affecting WT NPCs (Fig. 5g–j). We then validated the result of pharmacological inhibition by genetic knockdown of MDM2 using a small hairpin inhibitory RNA (*shMdm2*) (Fig. 5k–n). Hence, increasing the levels of HDAC1 protein through MDM2 inhibition also rebalances histone acetylation and rescues proliferation and differentiation deficits of *Fmr1* KO NPCs.

### Rebalancing histone acetylation rescues neurogenesis of *Fmr1* KO mice

**Fig. 6:**
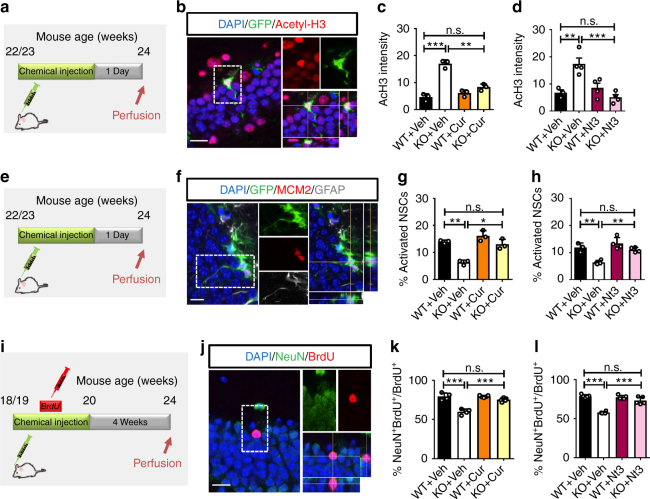
Rebalancing histone acetylation rescues neurogenic deficits in *Fmr1* KO mice. **a**, **e** Experimental schemes for assessing histone acetylation (**a**) and NSC activation (**e**) in WT and *Fmr1* KO mice treated with curcumin, Nutlin-3, or vehicle. **b** Sample confocal images of acetylated histone H3 in the NSCs of 6-month-old WT and *Fmr1* KO-Nes-GFP mice. Blue, DAPI; green, GFP; red, acetyl-H3. Scale bar, 20 µm. **c**, **d** Curcumin treatment (**c**, *n* = 3 mice per group) or Nutlin-3 (**d**, *n* = 3 to 4 mice per group) reduced histone H3 acetylation in GFP^+^ NSCs *Fmr1* KO mice without affecting WT mice. **f** Sample confocal images of activated NSCs (GFP^+^GFAP^+^MCM2^+^) in the dentate gyrus of 6-month-old WT or *Fmr1* KO (Nes-GFP) mice. Blue, DAPI; green, GFP; red, MCM2. Scale bar, 20 µm. **g**, **h** Curcumin (**g**, *n* = 3 to 4 mice per group) or Nutlin-3 treatment (**h**, *n* = 3 mice per group) rescued NSC activation in *Fmr1* KO mice. **i** Experimental scheme for assessing neuronal differentiation in WT and *Fmr1* KO mice treated with curcumin, Nutlin-3, or vehicle. **j** Sample confocal images of newborn neurons (DAPI^+^NeuN^+^BrdU^+^) in the dentate gyrus of WT and *Fmr1* KO mice. Blue, DAPI; green, NeuN; red, BrdU. Scale bar, 20 µm. **k**, **l** curcumin (**k**, *n* = 4 to 5 mice per group) or Nutlin-3 treatment (**l**, *n* = 4 to 5 mice per group) rescued neuronal differentiation specifically in *Fmr1* KO mice. *P < 0.05, **P < 0.01, ***P < 0.001, n.s.: no significant difference. Two-way ANOVA was used for all data analyses. Data are presented as mean ± s.e.m. The boxes with dotted white lines in **b**, **f**, **j** indicate regions where higher magnification images are shown

We next investigated whether rebalancing histone acetylation could rescue the neurogenic deficits in *Fmr1* KO mice. We decided to use curcumin instead of C646 to inhibit EP300 in vivo, because curcumin is already undergoing clinical trials for a large number of diseases^37^. In addition, curcumin is isolated from plants instead of being chemically synthesized, and therefore represents a potentially safe and low-cost option for future treatment. We also included a group of animals treated with Nutlin-3 as another way to rebalance histone acetylation through enhancing HDAC1. For this, we used a low-dosage Nutlin-3 treatment scheme we developed previously for young adult mice (10× lower dosage than used in cancer treatment)^14^. Previous studies have shown that both curcumin^41,42^ and Nutlin-3^43^ cross mouse blood–brain barrier within 60 min when given peripherally. Consistent with literature, we detected both curcumin (2.12 ng g^−1^ tissue) and Nutlin-3 (1297.03 ng g^−1^ tissue) at 60 min after a single injection at the dosage we planned to use (Supplementary Figs. 13, 14). Subsequently, we treated mice with either of these compounds at 5 months of age, when neurogenesis is actively declining^26,27^. We found that both curcumin and Nutlin-3 treatment reduced acetylation of histone H3 in Nestin-expressing cells to WT levels with no significant effect on WT mice (Fig. 6a–d). When analyzed at 1 day after curcumin or Nutlin-3 treatment, NSC activation in *Fmr1* KO mice was rescued to WT levels (Fig. 6e–h). In order to assess the fate of these NSCs, another cohort of mice received both chemical (curcumin or Nutlin-3) treatment and 5-bromo-2'-deoxyuridine (BrdU) injections to label dividing neural progenitors, which were analyzed at 4 weeks after the last treatment (Fig. 6i). We found that both curcumin and Nutlin-3 treatment enhanced neuronal differentiation in the DG of *Fmr1* KO mice and brought them to levels similar to those of WT littermate control mice (Fig. 6j–l). These results support a model in which curcumin or Nutlin-3 treatment rebalances histone acetylation and rescues the neurogenic deficits in mature adult *Fmr1* KO mice.

### Rebalancing histone acetylation rescues cognition of *Fmr1* KO mice

**Fig. 7:**
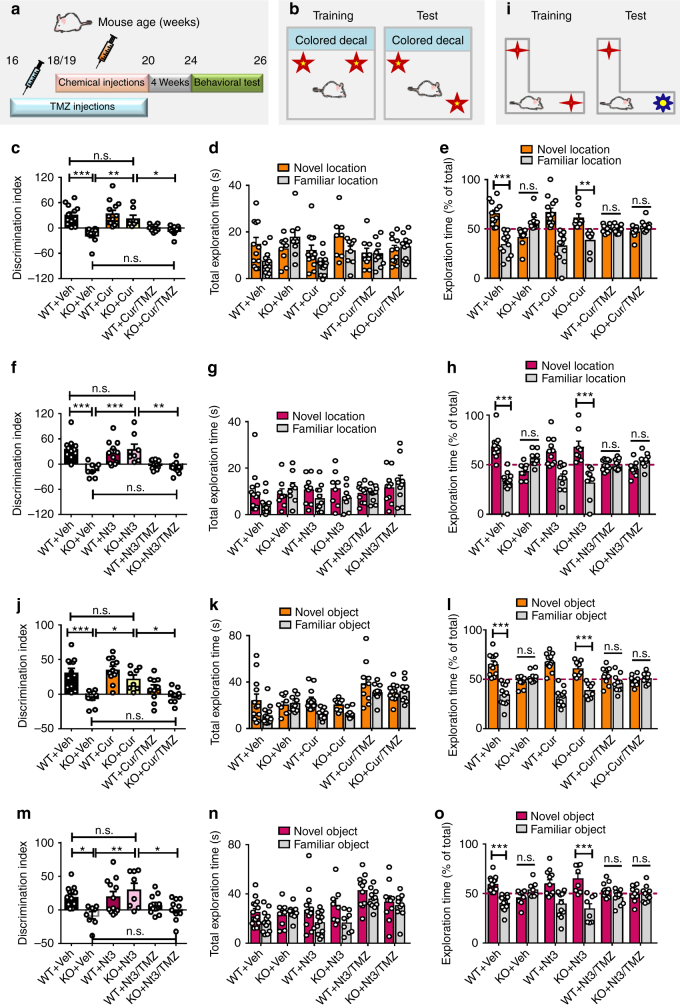
Rebalancing histone acetylation rescues cognitive deficits in 6-month old *Fmr1* KO mice. **a** Experimental scheme for assessing cognitive functions in WT and *Fmr1* KO mice with concurrent TMZ and various chemicals treatments. **b** Schematic of novel location test for assessing spatial learning. **c**–**h** Concurrent TMZ treatment prevents Curcumin- (**c**–**e**) (*n* = 8 to 13 mice per group) or Nutlin-3-induced (**d**–**h**) (*n* = 8 to13 mice per group) improvement of spatial memory in *Fmr1* KO mice in the novel location test. Total exploration time (**d**, **g**) and percentage exploration time (**e**, **h**) of novel location test are shown. **i** Schematic of the novel object recognition test. **j**–**o** Concurrent TMZ treatment prevents curcumin-(**j**–**l**) (*n* = 8 to 14 mice per group) or Nutlin-3-induced (**m**–**o**) (*n* = 8 to 12 mice per group) improvement of novel object recognition in *Fmr1* KO mice. Total exploration time (**k**, **n**) and percentage exploration time (**l**, **o**) of novel object recognition test are shown. *P < 0.05, **P < 0.01, ***P < 0.001, n.s.: no significant difference. Two-way ANOVA was used for all data analyses. Data are presented as mean ± s.e.m.

**Fig. 8:**
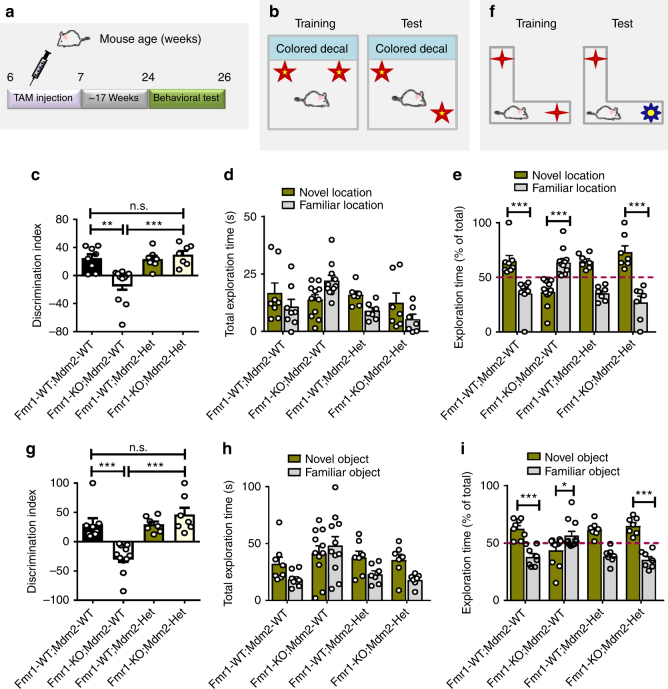
Heterozygote deletion of MDM2 in Nestin-expressing adult NSCs and new neurons rescues cognitive deficits of 6-month-old *Fmr1* KO mice. **a** Experimental scheme for assessing cognitive function transgenic mice followed by TAM injection. **b**–**e** Heterozygote deletion of MDM2 in Nestin-expressing adult NSCs and new neurons fully rescues spatial learning deficits in *Fmr1* KO mice (*n* = 7 to 11 mice per group). **f**–**i** Heterozygote deletion of MDM2 in Nestin-expressing adult NSCs and new neurons fully rescues novel object recognition in *Fmr1* KO mice (*n* = 7 to 11 mice per group). *P < 0.05, **P < 0.01, ***P < 0.001, n.s.: no significant difference. Two-way ANOVA was used for all data analyses. Data are presented as mean ± s.e.m.

It has been shown that age-dependent neurogenic decline is accompanied by age-associated decreases in spatial learning and memory in mice^26,27^. We therefore investigated whether rescuing adult neurogenesis is associated with restoring cognitive functions in mature adult *Fmr1* KO mice. Because NeuN-positive neurons in the DG and the cortex of *Fmr1* KO mice also exhibited elevated H3 acetylation compared to those in the WT mice, which could be corrected by curcumin or Nutlin-3 treatment (Supplementary Fig. 15), we decided to assess whether curcumin or Nutlin-3 treatment requires adult neurogenesis. We therefore treated some of the mice with temozolomide (TMZ), a DNA alkylating agent that has been used successfully to inhibit adult neurogenesis without significant effects on overall animal health and function^44,45^. We treated 5-month-old *Fmr1* KO mice and their WT littermates with TMZ prior to and during curcumin or Nutlin-3 treatment and assessed their cognitive functions using novel location test (NLT) and object recognition test (NOR) 4 weeks after the last treatment (Fig. 7a). Curcumin, Nutlin-3, or TMZ treatments had no significant effect on overall health, locomotor activity, or anxiety (Supplementary Fig. 16). *Fmr1* KO mice exhibited both impaired spatial learning in NLT and defective learning in a NOR compared to WT littermate control mice as shown in both reduced discrimination index and reduced percentage exploration time of the novel location/object (Fig. 7). Curcumin and Nutlin-3 treatments rescued deficits of *Fmr1* KO mice in both NLT (Fig. 7b–h) and NOR (Fig. 7i–o), while neither treatment affected WT mice. We found that inhibition of adult neurogenesis by TMZ abolished the rescue effect of both curcumin and Nutlin-3 (Fig. 7b–o). Therefore, the rescue effects of Nutlin-3 or curcumin require adult neurogenesis. To validate the results of TMZ, we created transgenic mice (*Nes-CreER*^*T2*^*;Fmr1-KO;Mdm2*^*f/w*^) that, upon tamoxifen injections, led to heterozygote deletion of *Mdm2* gene specifically in adult NESTIN-expressing NSCs and their progenies in *Fmr1 KO* mice (Supplementary Fig. 17a, b). Genetic reduction of MDM2 in adult new neurons did not affect overall locomotor activity or anxiety in either WT or *Fmr1* KO mice (Supplementary Fig. 17c, d) but rescued behavioral deficits of *Fmr1* KO mice (Fig. 8).

**Fig. 9:**
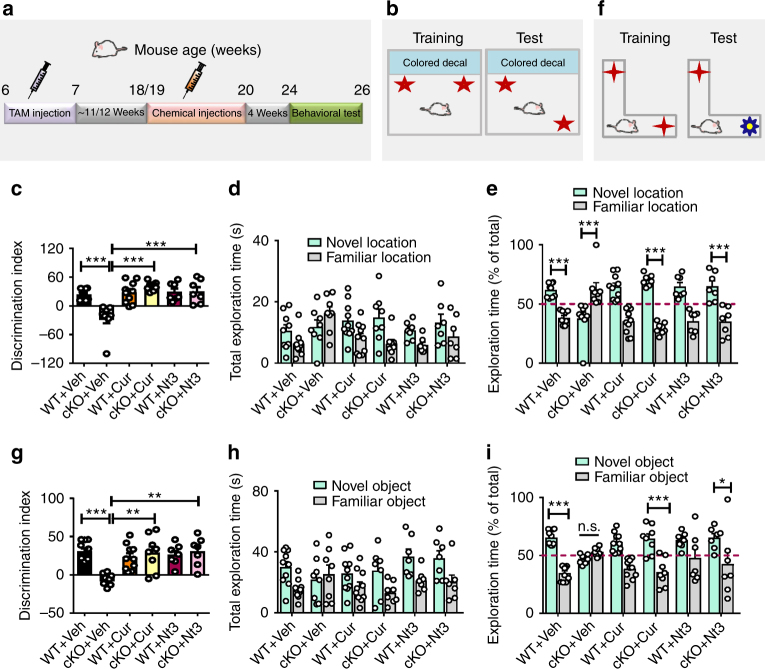
Rebalancing histone acetylation rescues cognitive deficits in 6-month-old *Fmr1 cKO;Cre;Ai14* mice. **a** Experimental scheme for assessing cognitive functions in mice and *Cre;Ai14* control mice, followed by TAM injection and various chemical treatments. **b**–**e** Curcumin or Nutlin-3 treatment rescues spatial learning deficits in *Fmr1 cKO;Cre;Ai14* as mice determined by novel location test (*n* = 7 to 10 mice per group). Total exploration time (**d**) and percentage exploration time (**e**) of novel location test are shown. **f**–**i** Curcumin or Nutlin-3 treatment fully rescues deficits in the novel object recognition in *Fmr1 cKO;Cre;Ai14* mice (*n* = 7 to 10 mice per group). Total exploration time (**h**) and percentage exploration time (**i**) of novel object recognition test are shown. *P < 0.05, **P < 0.01, ***P < 0.001, n.s.: no significant difference. Two-way ANOVA was used for all data analyses. Data are presented as mean ± s.e.m.

We have previously shown that selective deletion of FMRP in adult new neurons lead to impaired neurogenesis and behavioral deficits in young adult mice^12,14^. Since *Fmr1* cKO mice that had tamoxifen-induced FMRP specifically deleted in adult NSCs exhibited impaired neurogenesis at 6 months of age (Fig. 1i–l and Supplementary Fig. 2), we treated these mice with curcumin or Nutlin-3 at 5 months of age. Mice with selective deletion of FMRP from NSCs and their progenies exhibited cognitive deficits in both NLT and NOR tests, which were rescued by either curcumin or Nutlin-3 treatments (Fig. 9). Taken together, these results suggest that cognitive rescue of *Fmr1* KO mice by curcumin and Nutlin-3 requires adult hippocampal neurogenesis.

## Discussion

Although FXS is categorized as a neurodevelopmental disorder, FXS patients have a normal lifespan, and their neurological symptoms and cognitive deficits persist throughout adult life into old age. However, most research and interventions have been focused on developing and young adult brains. In addition, although many FMRP candidate targets have been identified^2,3^, few have been validated for their biological functions, let alone therapeutic targets. We unveil a novel function of FMRP in balancing histone acetylation through the regulation of both histone acetylation and deacetylation pathways. We show that rebalancing histone acetylation using clinical trial compounds, particularly curcumin, rescues the cognitive functions of FXS mice. These exciting results represent a novel treatment strategy for FXS, especially for mature adult individuals with FXS.

Histone acetylation plays important roles in chromatin structure and gene regulation. Altered histone acetylation has been found in a number of diseases, including both psychiatric disorders and neurological disorders^22^. We found that FMRP deficiency led to elevated histone H3 and H4 acetylation in NPCs and neurons, which is consistent with a recent finding^24^. Histone acetylation is controlled by balanced activity of histone acetyltransferases (e.g. EP300) and HDACs. Interestingly, our data show that a lack of functional FMRP leads to both elevated EP300 and reduced HDAC1 in NPCs, which significantly tilted the balance of histone acetylation. Both EP300 and HDAC1 seem to play important roles in adult brain plasticity and cognitive functions. Although forebrain-specific deletion of EP300 impairs memory formation^46^, elevated EP300 has been associated with impaired memory and inhibition of EP300 restores memory formation^47^. In addition, EP300 activates transcription of P16, a CDK4/6 inhibitor^30,48^ which exhibited increased levels in *Fmr1* KO NPCs (Supplementary Fig. 6). Elevated P16 levels can lead to cell cycle arrest and neurogenesis decline^30,31^ and are associated with aging and cognitive impairment^49^. Among the large number of HDACs in mammals, HDAC1 is expressed highly in NSCs and plays an important role in maintaining NSC self-renewal during brain development^50,51^. Acute knockdown of HDAC1 in adult hippocampus also leads to impaired contextual fear memory^52^. The age-dependent decline of the HDAC1-containing NuRD complex has been associated with cognitive decline^53^. In the absence of FMRP, a combination of elevated EP300 and reduced HDAC1 lead to drastic increase in histone acetylation in NPCs, which can have significant impact on gene expression. Although we did not observe altered EP300 or HDAC1 in hippocampal and cortical neurons of 6-mo *Fmr1* KO mice, we did find elevated H3 acetylation in DG and cortical neurons in 6-mo *Fmr1* KO mice, which could result from alteration of other epigenetic regulators as demonstrated recently^24^. The postmortem hippocampal tissues from two patients with FXS also exhibited higher histone acetylation levels compared to those from age- and gender-matched healthy controls (Supplementary Fig. 18), suggesting that elevated histone acetylation might be a hallmark of FMRP deficiency. A comparison of mRNAs that are dysregulated in NPCs differentiated from human FXS patient-derived pluripotent stem cells^54^ and genes bound by acetyl-histone H3 in mouse cortex (https://www.encodeproject.org/experiments/ENCSR000CDD/) show a significant overlap (Supplementary Fig. 19). Our discovery of elevated histone acetylation in FXS opens up new possibilities for drug repurposing by targeting enzymes controlling histone acetylation.

As an EP300 inhibitor^35^, curcumin is able to reverse impaired hippocampal neurogenesis and cognition in animals^55^. Since our goal was to correct NSC activation and avoid potential side effects as much as possible, we chose a shortened injection period^56^ and a significantly lower (10–20×) dosage than what has been used previously to modulate neurogenesis^38^. We found that a low dose of curcumin rebalanced histone acetylation and rescued neurogenic and cognitive deficits in adult FXS mice, which may extend the application of curcumin to the single-gene etiology of autism and provide a hope for FXS patients and their families by mitigating the high monetary- and time-related costs and risks of treating disease symptoms. Nutlin-3 is the prototype MDM2 inhibitor and has been used in clinical trials for cancer^39,57^. The fact that both curcumin and Nutlin-3 are effective for treating 6-mo mice suggests a potential therapeutic opportunity for mature adult and aging FXS patients, which are largely understudied. For treating brain disorders, a significantly lower dosage of Nutlin-3 and curcumin than those used for cancer treatment should be considered.

Our studies characterize an important role of FMRP in cognitive maintenance in mid-age mature adults through preserving adult NSC pools, which has not been shown before. Our previous series of experiments showed that FMRP deficiency led to increased NSC activation through the MDM2–P53 pathway in 2-month-old (young adult) FMRP-deficient mice^14^. Here we show that a loss of FMRP leads to depletion of NSCs and decreased NSC activation when mice reach 6 months of age (mid-age). A remaining question is why these two populations of NSCs exhibit differential changes upon FMRP deficiency. It is possible that when the NSC population diminishes significantly, NSCs changes their responses to regulatory mechanisms. Another possibility is that NSCs have age-specific intrinsic regulatory programs, as demonstrated in both hematopoietic stem cells and NSCs^58^. An age-dependent decline of the NuRD complex (containing HDAC1) has been associated with cognitive decline, which can be rescued by expressing NuRD components in aged brains^53^. It is likely that FMRP deficiency exaggerated these age-dependent changes. Since 6 months of age in mice is roughly analogous to middle age in humans, we are not investigating the full extent of aging per se. It would be valuable to evaluate these animals at 12, 18, and 24 months of age. In our preliminary analysis of 6-mo and 12-mo mice, we found that adult hippocampal neurogenesis in mice already reached low levels at 6 months, and it becomes difficult to discern any further differences between 6-mo and 12-mo mice. Therefore, we used 2-month and 6-month time points to study the temporal dynamics of NSC activity in the present study. When non-invasive methods become more readily available, it will be important to evaluate adult neurogenesis in aging and aged FXS and healthy human populations. In addition to P53 and HDAC1, MDM2 may target other proteins in an age-specific manner. However, a comprehensive analysis of MDM2 cofactors or targets have not been done in the nervous system, which we hope to initiate based on the present study. Our results underscore the importance of FMRP in cognitive maintenance in adult mammals, raising the question of FMRP function in aging populations.

**Fig. 10:**
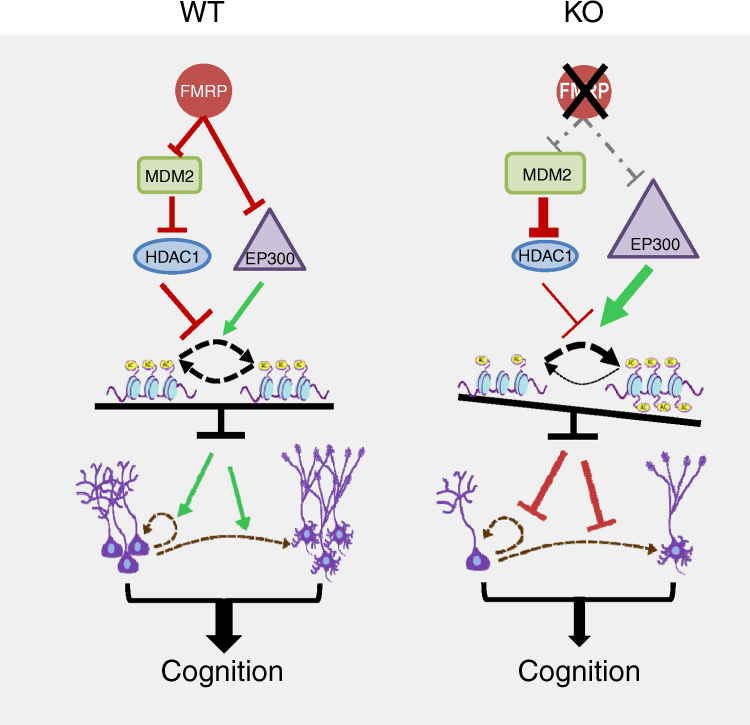
Models for FMRP maintain histone acetylation through regulating both EP300 and HDAC1 expression levels, which impact adult neurogenesis and cognition

In summary, our data show that the clinical drugs can reverse the neurogenic and cognitive deficits of mature adult FXS mouse models through the rebalancing of histone acetylation (Fig. 10). This observation not only provides a potential new treatment for adult FXS patients, but also for a broader range of neurological and neuropsychiatric disorders. We did not observe significant changes in protein levels of HDAC1 or GSK3β in 6-mo *Fmr1* KO hippocampal or cortical tissues, with or without curcumin or Nutlin-3 treatment (Supplementary Fig. 20). However, total protein levels do not reflect changes in activities. For example, elevated GSK3β activities, but not total protein levels, have been found in the DG but not the CA1 sub-regions of the hippocampus^59^. In fact, GSK3β inhibitors can rescue cognitive deficit of Fmr1 KO mice^59–61^. In addition, it remains possible that, when treated at young age especially juvenile period, curcumin and Nutlin-3 may still exert effects through targeting these molecules and through other cell types instead of adult new neurons, which may rescue adult neurogenesis-independent behavioral deficits found in juvenile mice, such as hyperactivity and social interaction, etc. Finally, FMRP is predicted to regulate a large number of targets and a number of treatments towards these targets may restore cognitive function in mouse models. There is likely a convergence of multiple pathways that can be manipulated by targeting each upstream component. A study of a shared downstream effector pathways should be performed for a better understanding of FMRP functions and therapeutic development.

## Methods

### Animal studies

All animal procedures were performed according to protocols approved by the University of Wisconsin-Madison Care and Use Committee. All mice were on C57B/L6 genetic background. The *Fmr1* KO;Nestin-GFP mice (*Fmr1*^*−/y*^*;Nestin-GFP*) were created by crossing female *Fmr1* heterozygous KO mice (*Fmr1*^*+/−*^)^62^ with homozygous Nestin-GFP transgenic males^63^. Generation of FMRP inducible conditional mutant mice (*Fmr1*^*loxP/y*^*;Nestin-CreER*^*T2*^*;Rosa26-tdT, or cKO;Cre;Ai14*) and tamoxifen injections to induce recombination were performed as previously described^64^. To induce recombination, mice (6 weeks old) received Tamoxifen (Sigma-Aldrich) daily for 5 days (160 mg kg^−1^) as previously described^14^. Nutlin-3 (8 mg kg^−1^) or curcumin (0.4 mg kg^−1^) was given to 22–23-week-old mice through intraperitoneal (i.p.) injections every other day (for Nutlin-3 treatment)^14^ or every day (for curcumin treatment)^56^ for 5 injections and killed at 24 h after the last injection for NSC proliferation analysis. Generation of MDM2 inducible conditional knockdown mice that are either *Fmr1*-KO or WT (*NesCre;Fmr1*^*+/y*^*;Mdm2*^*f/w*^ *or NesCre;Fmr1*^*−/y*^*;Mdm2*^*f/w*^) and tamoxifen injection to induce recombination were performed as previously described^12^. For NSC differentiation analysis, the same dosage of Nutlin-3 or curcumin was given to 18–19-week-old mice through i.p. injections using the same procedures as described above and killed at 4 weeks after the last injection. Meanwhile, mice also received four BrdU injections (100 mg kg^−1^) between days 2 and 3 of Nutlin-3 or curcumin injections. To repress endogenous neurogenesis, 16–17-week-old mice received i.p. injection of TMZ (50 mg kg^−1^, Selleckchem, S1237) during the first 3 days of each week for 4 weeks as previously described^44^. Behavioral testing started 4 weeks after the final TMZ injection. Data collection was carried out for a predetermined period of time, as dictated by literature- or core facility-based standards, and no exclusion criteria were applied. Because *Fmr1* is an X-linked gene and *Fmr1* KO male mice have significantly reduced fertility, we used only male littermate WT and *Fmr1* KO mice for our study. For the analysis of the chemical concentration in the brain, Nutlin-3 (8 mg kg^−1^) or curcumin (0.4 mg kg^−1^) was injected i.p. into three B57BL/6 mice. The animal was killed at 60 min after a single chemical injection, and the brain was removed, snap frozen in liquid nitrogen, and stored at −80 °C until analysis. For drug treatment, animals were randomly assigned to treatment arms with approximately equivalent numbers in each group. All cell counting and behavioral analyses were performed by experimenters who were blind to the identity and treatments of the sample.

### Tissue preparation and immunohistochemistry

Brain tissue processing and histological analysis of mouse brains were performed as described in our publications^12,14^. Mice were transcardially perfused with saline followed by 4% paraformaldehyde (PFA). Brains were post-fixed overnight in 4% PFA and 40 μm brain sections were used for immunohistological staining. The primary and secondary antibodies used are described in Supplementary Methods.

### In vivo cell quantification

The total numbers of GFP^+^ or MCM2^+^ cells in the DG of each animal were determined by unbiased stereology methods using Stereo Investigator software (MBF Biosciences) as previously described^14^. Z-stack images (2 μm interval) were acquired using an AxioImager Z2 ApoTome confocal microscope (Plan-APOCHOROMAT, 20×, numerical aperture = 0.8; Zeiss). The cell numbers were quantified by counting one in six coronal serial sections containing the hippocampus. Schaeffer’s coefficient of error was <0.1 for each type of cell quantification. The experimenter was blinded to the identity of the samples. Cell-lineage analysis was performed as previously described^14^. At least 50 GFP^+^ or BrdU^+^ cells in the DG were randomly selected, and their co-localization with cell-lineage markers was determined using a Nikon A1 confocal microscope and quantified using Image J software (NIH). DG volume was determined by the Stereo-Investigator software by summing the traced granule cell areas for each section multiplied by the distance between sections sampled as described^14^.

The signal intensity of acetyl-H3 in Nestin GFP^+^ cells in the DG or in NeuN^+^ cells in the DG and cortex (layer II/III) of each animal was quantified using Image J software as previously described^65^. The z-stack images (2 μm interval) were acquired using Nikon A1 confocal microscope. At least 10 individual GFP^+^ or NeuN^+^ cells were randomly selected from DG or cortex (layer II/III) of the brain sections in each animal and the fluorescent intensity of acetyl-H3 was measured after subtracting background pixel intensity in the same image using Image J software (NIH). The average intensity from each animal (at least 10 cells) was count as *n* = 1 for statistical analysis. Samples from three to four individual animals, each from a different litter, per experimental condition were analyzed (*n* = 3 or 4).

### Adult NPC analyses

NPCs were isolated from the DG of 6-month-old male *Fmr1* KO mice and WT littermate controls based on our published method^66^. Independently isolated cells serve as biological replicates. Proliferation and differentiation of NPCs were analyzed as previously described^14^. We used only early passage cells (between passages 4 and 10) and only the same passage numbers of WT and *Fmr1* KO cells. For each experiment, triplicate wells of cells were analyzed, and results were averaged as one data point (*n* = 1). At least 3 independent biological replicates were used (*n* = 3) for statistical analyses.

### Lentivirus expressing shRNA

The shRNA against mouse MDM2 has been previously published^14^. ShRNA against mouse EP300 (5′-GCACAAATGTCCAGTTCTTCT-3′) was designed based on mouse *Ep300* mRNA sequence. ShEP300 was then cloned into the *Hpa*I and *Cla*I sites of a lentivector using lentivirus-U6 promoter-miR137-CMV promoter-GFP vector as the backbone, according to our published method^67^. The shNC sequence (5′-GGAATCTCATTCGATGCATAC-3′) has been published previously. All plasmid construct was verified by DNA sequencing.

### Chemical treatment

For proliferation assay in vitro, 0.5 μM curcumin (Selleckchem, S1848), 0.8 μM C646 (Selleckchem, S7152) (EP300 inhibitor), or 0.8 μM Nutlin-3 (Nt3, Selleckchem, S1061) (Mdm2 inhibitor) was added to proliferating NPCs at 6 h post plating. Cells were then pulse labeled 18 h later with 5 μM BrdU (Sigma-Aldrich, B5002) and incubated for 6 h in the presence of chemical treatment, followed by fixation, staining, and coverlipping. For differentiation assay in vitro, 0.5 μM curcumin, 0.8 μM C646, or 0.8 μM Nt3 was added to aNPCs upon initiation of differentiation together with RA/Fsk for 4 days, followed by fixation, staining, and coverlipping. The quantitative analysis of number of BrdU- or Tuj1-expressing cells among total (DAPI^+^ nuclei) were quantified using unbiased stereological method (MBF Biosciences).

### RNA immunoprecipitation

RNA-IP was performed as previously described^14^. A monoclonal antibody against FMRP (7G1-1, DSHB) was used to immunoprecipitate FMRP and its bound mRNA. The precipitated mRNA was resuspended into Trizol (Invitrogen) for RNA isolation. Real-time PCR was performed using standard methods as previously described^14^. The primer sequences and calculation are provided in Supplementary Methods.

### Real-time PCR assay

Real-time PCR was performed using standard methods as previously described^14^. The first-strand complementary DNA (cDNA) was generated by reverse transcription with Oligo (dT) primer (Roche). To quantify the mRNA levels using real-time PCR, aliquots of first-stranded cDNA were amplified with gene-specific primers and Power SYBR Green PCR Master Mix (Bio-Rad) using a Step-1 Real-Time PCR System (Applied Biosystems). The PCR reactions contained 1 μg of cDNA (except the cDNA for the IP, for which 5% of the cDNA was used for each gene examined), Universal Master Mix (Applied Biosystems), and 10 μM of forward and reverse primers in a final reaction volume of 20 μL. The mRNA level of different samples was calculated by the data analysis software built in with the 7300 Real-Time PCR System. For RIP-real-time PCR, cDNA from IP and input was used and IP samples were normalized to Input samples. The Sequences of primers are provided in Supplementary Methods.

### Bio-orthogonal non-canonical amino acid tagging (BONCAT)

BONCAT experiment was carried out as previously described^29^. NPCs were starved in conditional medium without Methionine for 1 h. Azidohomoalaine (AHA), an analog of Methionine, was added into NPCs and incorporated into nascent proteins for 2 h. Afterward, WT and *Fmr1* KO NPCs were harvested using lysis buffer (10 mM Tris pH 7.5, 1% SDS) with 2× EDTA-Free protease inhibitor cocktails (Roche). The azide enables conjugation to beads for enrichment and purification. Copper-catalyzed azide-alkyne cycloaddition was used to conjugate Biotin alkyne to nascent proteins in the whole protein lysates and were then diluted with phosphate-buffered saline (PBS) to a final volume of 1 mL and excess biotin was removed by dialysis against PBS overnight at 4 °C. AHA-biotin-tagged proteins were precipitated using Streptavidin Agarose resin (Thermo Scientific, IL, USA) overnight at 4 °C. After three washes with lysis buffer, the precipitates were then separated on an sodium dodecyl sulfate–polyacrylamide gel electrophoresis (SDS-PAGE) for protein analysis and probed with specific antibodies.

### Protein stability analysis

Protein stability analysis was carried out as previously described^33^. NPCs from WT and *Fmr1* KO mice were grown in proliferating condition. Cycloheximide (Sigma-Aldrich) was added to culture medium at 50 μg mL^−1^. Cells were collected at different time points (from 0 h to 8 h) of treatment. The cells were then lysed and 20 μg of proteins were loaded to each lane for western blotting analysis using an antibody against HDAC1. The relative amount of protein remain was calculated by comparing to time point 0.

### Co-immunoprecipitation (Co-IP) assay

Co-IP experiment was performed according to the manufacturer’s instructions of Pierce Co-IP kit (Thermo Scientific, #26149). NPCs were harvested and homogenized in ice-cold lysis buffer (25 mM Tris, 150 mM NaCl, 1 mM EDTA, 1% NP-40 and 5% glycerol; pH 7.4) with 2× complete protease inhibitor cocktails (Roche). Nuclei and debris were pelleted at 10,000 × *g* for 20 min. The supernatant was collected and the protein concentrated was quantified. The resulting supernatant was pre-cleared for 1 h with Pierce Control Agarose Resin. A monoclonal antibody against HDAC1 (BioVersion, 3601-30) or MDM2 (Santa Cruz, sc-965) was incubated with AminoLink Plus coupling Resin for 2 h and washed 3 times with coupling buffer (10 mM sodium phosphate, 150 Mm NaCl; pH 7.2). Meanwhile, a monoclonal antibody against IgG (Cell signaling, #5415s) was utilized as a negative control. The pre-cleared lysates (1 mg of proteins) were immunoprecipitated with antibody-coated Resin at 4 °C for 2 h. After three washes with lysis buffer, the precipitates were then separated on an SDS-PAGE for protein analysis and probed with anti-MDM2 or anti-HDAC1 antibody, respectively.

### GST-labeled UBD pull down

Tandem human ubiquitin-binding domains (THUBDs) consisting of GST tag and two tandem UDA20s were purified as previously described^34^, THUBDs were overexpressed in *Escherichia coli* BL21 (DE3) cells, and the harvested cells were lysed by sonication in lysis buffer (1 mM dithiothreitol, 1% Triton X-100 in PBS). THUBDs were purified from cell lysates using glutathione-Sepharose (GSH) 4B beads (Qiagen, Valencia, CA) according to the manufacturer’s instructions. For GST pull-down assay, lentivirus-shMdm2-GFP or lentivirus-shNC-GFP were infected and maintained for 2 days in NPCs, respectively. Cells were treated with MG132 (25 µg mL^−1^) 6 h before collecting samples. Cell lysates were collected in ice-cold lysis buffer (Tris-HCl pH 8.0, 150 mM NaCl, 1% NP-40, 10 mM IAA, 0.01% SDS) with 2× complete protease inhibitor cocktails (Roche). GST-UBD was loaded on Sepharose 4B affinity beads overnight at 4 °C. After washing the coupled beads with lysis buffer 3 times, the cell lysates incubated with the coupled beads at 4 °C for 30 min. After removal of supernatant, the coupled beads were washed 3 times with lysis buffer. The precipitates were then separated on an SDS–polyacrylamide gel electrophoresis for protein analysis and probed with anti-HDAC1 antibody.

### Western blotting analyses and ELISA

Protein samples were separated on SDS-PAGE gels (Bio-Rad), transferred to polyvinylidene difluoride membranes (Millipore), and incubated with primary antibodies. The information for antibodies are provided in the Supplementary Methods. Enzyme-linked immunosorbent assay (ELISA) for EP300 was performed using a commercially available kit (USCN Life Science Inc., San Diego, CA) according to the manufacturer’s instruction. Full size blots are shown in Supplementary Figs. 21–27.

### Novel location test

This test measures spatial memory through an evaluation of the ability of mice to recognize the new location of a familiar object with respect to spatial cues. The experimental procedure was developed by the core facility of Waisman Center at UW-Madison, based on the description for a Novel Object exploration in mice^68^. Mice were handled for approximately 5 min a day for a maximum of 5 days prior to the experiment. Testing consisted of five 6 min trials, with a 3 min intertrial interval between each trial. All procedures were conducted during the light cycle of the animal between 10 a.m. and 4 p.m. Before the trial session, mice were brought into testing room and were allowed to acclimate for at least 30 min. During the intertrial interval, the mouse was placed in a holding cage, which remained inside the testing room. In the first trial (Pre-Exposure), each mouse was placed individually into the center of the otherwise empty open arena (38.5 cm Long × 38.5 cm wide, and 25.5 cm high walls) for 6 min. For the next three trials (Sample Trials 1–3), two identical objects were placed equidistantly from the arena wall in the corners against the wall with the colored decal. Tape objects to the floor of the arena. Then, each mouse was placed individually into the center of the arena, and was allowed to explore for 6 min. At the end of the trial, the mouse was removed and returned to the home cages for 3 min. In the last trial (Test), one of the objects was moved to a novel location, and the mouse was allowed to explore the objects for 6 min, and the total time spent exploring each object was measured. During the test phase, exploration time was defined as any investigative behavior (i.e., head orientation, climbing on, sniffing occurring within <1.0 cm) or other motivated direct contact occurring with each object. To control for possible odor cues, objects were cleaned with 70% ethanol solution at the end of each trial and the floor of the arena wiped down to eliminate possible scent/trail markers. During the test phase, two objects were wiped down prior to testing so that the objects would all have the same odor. Based on a study^69^, the discrimination index was calculated as the percentage of time spent investigating the object in the new location minus the percentage of time spent investigating the object in the old location: discrimination index = (novel location exploration time/total exploration time × 100)−(old location exploration time/total exploration time × 100). A higher discrimination index is considered to reflect greater memory retention for the novel location object. All experiments were videotaped and scored by scientists who were blinded to experimental conditions to ensure accuracy.

### Novel object recognition test

This test is based on the natural propensity of rodents to preferentially explore novel objects over familiar ones. Mice were handled for approximately 5 min a day for a maximum of 5 days prior to the experiment. The experimental procedure was performed as previously described^70^ and was conducted during the light cycle of the animal between 10 a.m. and 4 p.m. Before the trial or test phase, mice were brought into testing room and were allowed to acclimate for at least 30 min. On the first day, mice were habituated for 10 min to the V-maze, made out of black Plexiglas with two corridors (30 cm long × 4.5 cm wide, and 15 cm high walls) set at a 90° angle, in which the task was performed. On the second day, mice were put back in the maze for 10 min, and two identical objects were presented. After 24 h, one of the familiar objects was replaced with a novel object, and the mice were again placed in the maze and were allowed to explore for 10 min, and the total time spent exploring each of the two objects (novel and familiar) was measured. During the test phase, the novel and familiar objects were wiped down prior to testing so that the objects would all have the same odor and exploration time was defined as the orientation of the nose to the object at a distance of less than 2 cm. The discrimination index was calculated as the difference between the percentages of time spent investigating the novel object and the time spent investigating the familiar objects: discrimination index = (novel object exploration time/total exploration time × 100)−(familiar object exploration time/total exploration time × 100). A higher discrimination index is considered to reflect greater memory retention for the novel object. All experiments were videotaped and scored by scientists who were blinded to experimental conditions to ensure accuracy

### Statistical analysis

All experiments were randomized and blinded to scientists who performed quantification. Statistical analysis was performed using analysis of variance (ANOVA) and Student’s *t*-test, unless specified, with the GraphPad Prism software. Two tailed and unpaired *t*-test was used to compare two conditions. Two-way ANOVA was used for comparison among multiple experimental conditions. Tukey's post hoc test was used when comparing among each condition. For HDAC1 protein degradation assay, two-way ANOVA was used to evaluate protein decay rates. All data were shown as mean with standard error of mean (mean ± s.e.m). Probabilities of *P* < 0.05 were considered as significant.

### Data availability

All data generated for this study are available from the corresponding authors upon reasonable request.

## Electronic supplementary material

Supplementary Information

Peer Review File

Description of Additional Supplementary Files

Supplementary Data 1
