# Supplementary material for: Reducing histone acetylation rescues cognitive deficits in a mouse model of Fragile X syndrome
Source: Nat Commun. 2018 Jun 27;9:2494. doi: 10.1038/s41467-018-04869-3 (PMC6021376; doi:10.1038/s41467-018-04869-3)
Supplement: Supplementary file 1 — Supplementary Information [file 41467_2018_4869_MOESM1_ESM.pdf]

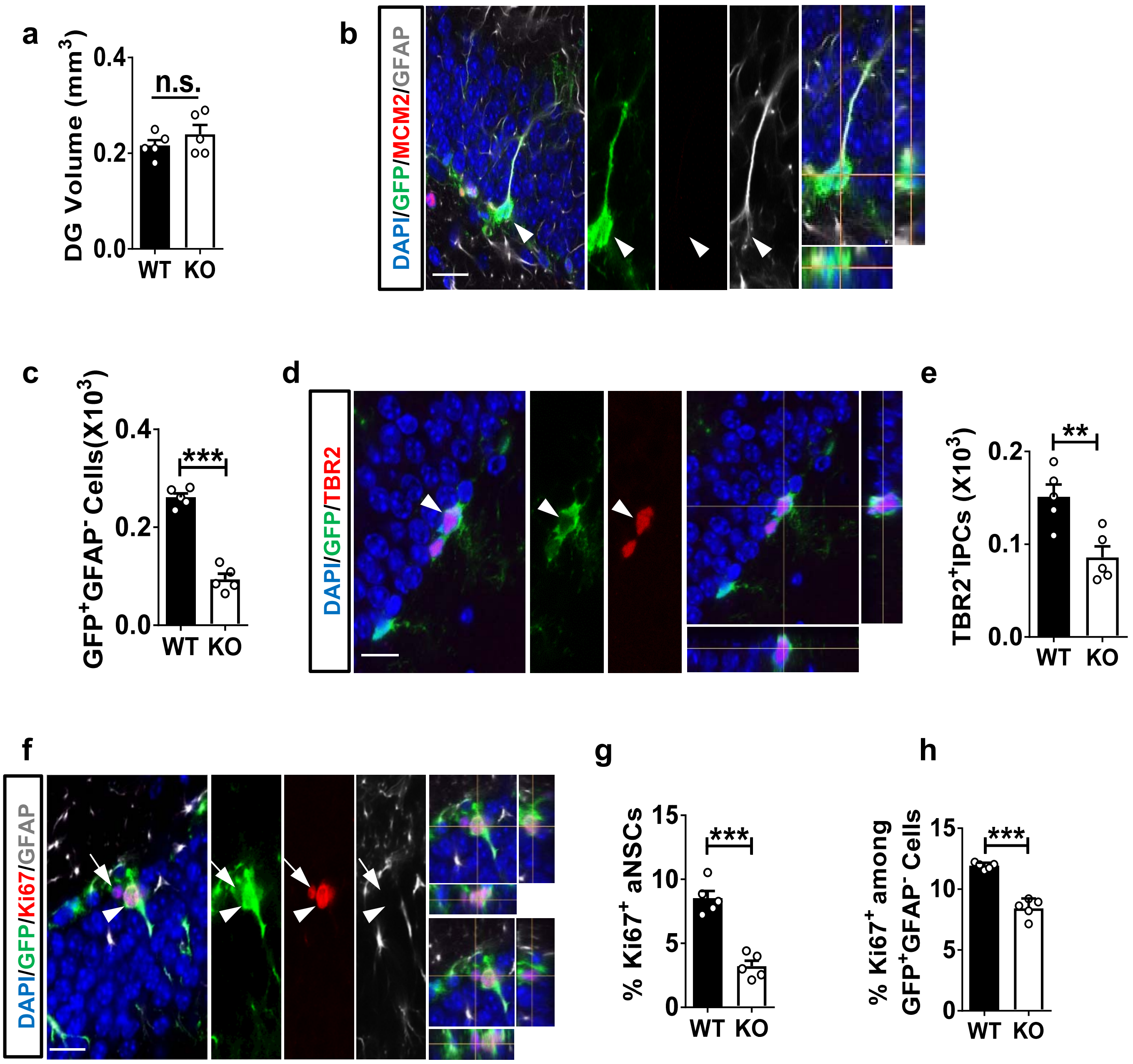

Supplementary Figure 1: FMRP deficiency does not affect dentate gyrus volume but leads to changes in the numbers of activated NSCs in 6-month old WT and *Fmr1* KO mice.

(**a**) Quantitative comparison of dentate gyrus volume of adult *Fmr1* KO mice and wild-type (WT) littermate controls. (**b**) Sample confocal image of $MCM2^-$ cells in the dentate gyrus of adult mice. White arrowheads, quiescent NSCs. Scale bar, 20μm. (**c**) Quantitative comparison of $MCM2^+$ cells in the dentate gyrus of adult *Fmr1* KO mice and wild-type (WT) littermate controls, (n = 5 per genotype). **P<0.01, ***P<0.001, n.s., no significant difference. Student's t-tests were used for data analyses. Data are presented as mean ± s.e.m.

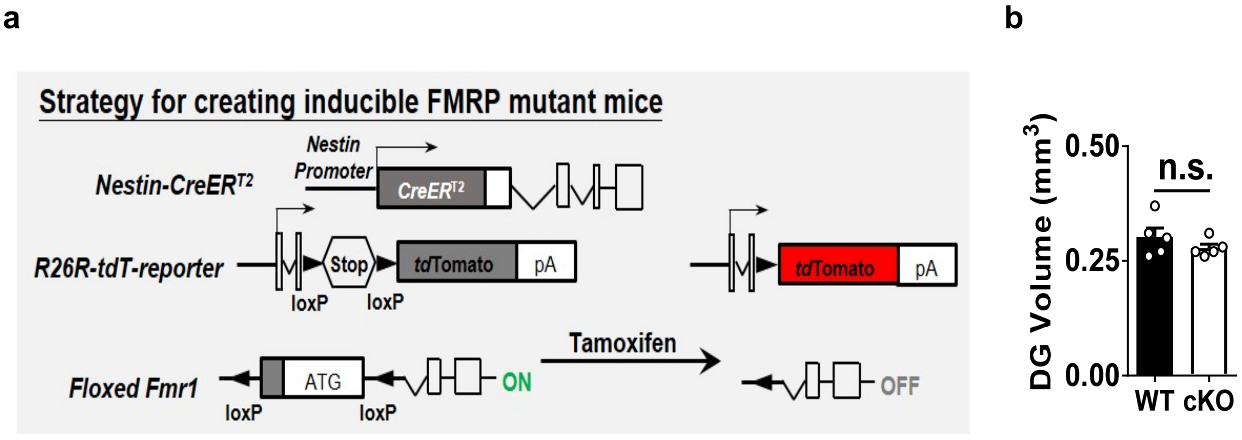

Supplementary Figure 2: Conditional deletion of FMRP did not affect dentate gyrus volume.

(**a**) An inducible FMRP conditional knockout mouse line was created by crossing *Nestin-CreER*^{T2} (Cre) mice, *ROSA26-STOP-tdTomato* (Ai14) mice and *Fmr1* floxed (*Fmr1* cKO) mice. Administration of tamoxifen to adult mice results in the removal of the first exon of the mouse *Fmr1* gene and the “Stop” codon before tdTomato (tdT) in Nestin-expressing cells and their subsequent progenies. (**b**) Quantitative comparison of dentate gyrus volume of *Cre;cKO; Ai14* (cKO) mice and *Cre;Ai14* (WT) control mice (n=5 per genotype). n.s., no significant difference. Student’s t-tests. Data are presented as mean$\pm$ s.e.m.

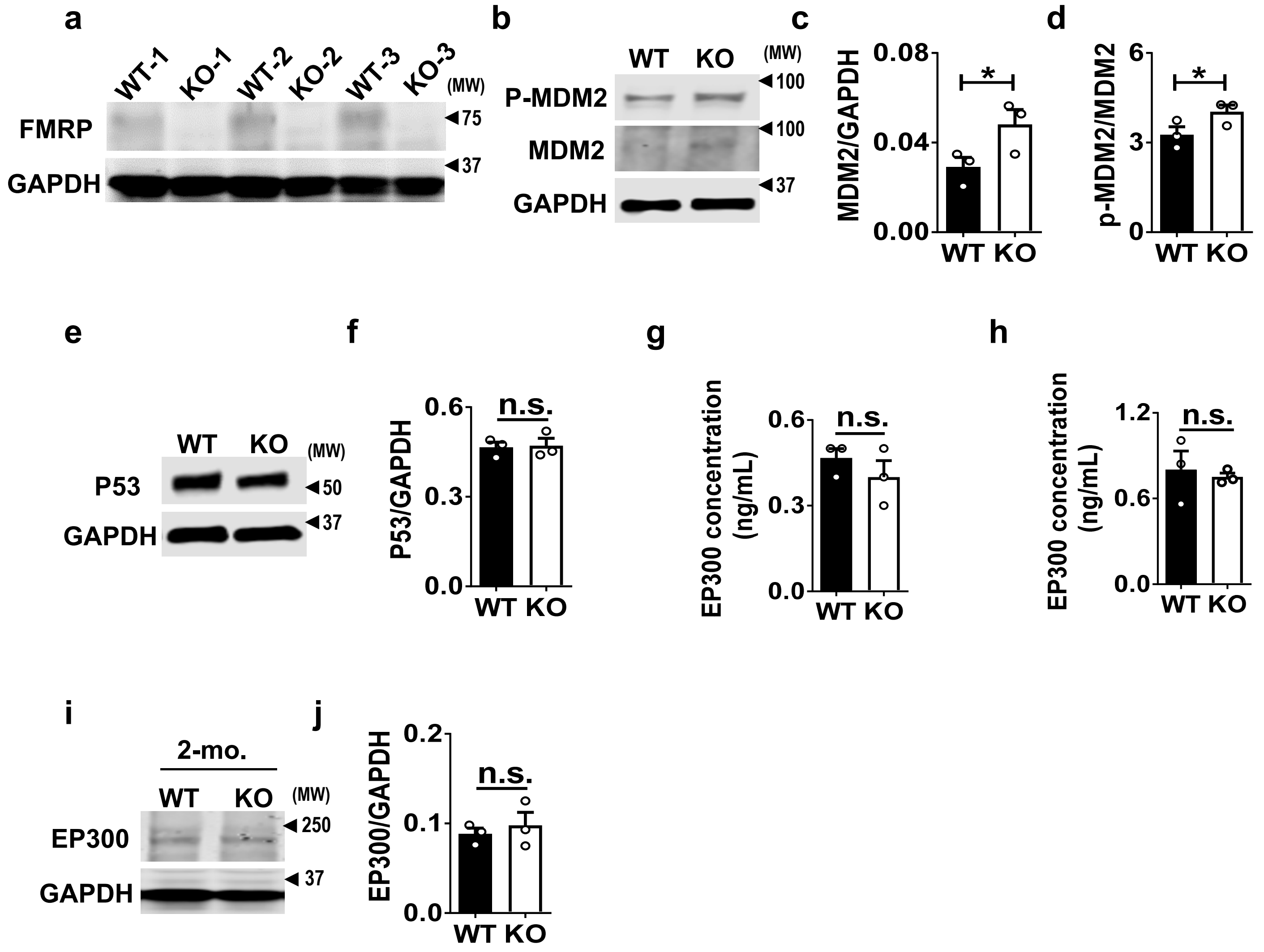

Supplementary Figure 3: Western blot or ELISA analyses of protein levels in NPCs or brain tissues of WT and *Fmr1* KO mice.

(a) FMRP protein was detected in primary NPCs independently isolated from three WT mice but was not detected in those isolated from *Fmr1* KO mice. (**b-d**) Total MDM2 and phosphorylated MDM2 at Ser-166 (P-MDM2) in 6-mo WT and *Fmr1* KO NPCs, (n=3). (**e, f**) P53 levels in 6-mo WT and *Fmr1* KO NPCs, (n=3). (**g, h**) EP300 protein levels were assessed by ELISA in the hippocampus (**g**) and cortex (**h**) of 6-mo WT and *Fmr1* KO mice, (n=3 per genotype) (**i, j**) EP300 protein levels in 2-mo NPCs. (n=3) GAPDH was used as a loading control. *P<0.05, n.s., no significant difference. Student's *t*-tests were used for data analyses. Data are presented as mean ± s.e.m.

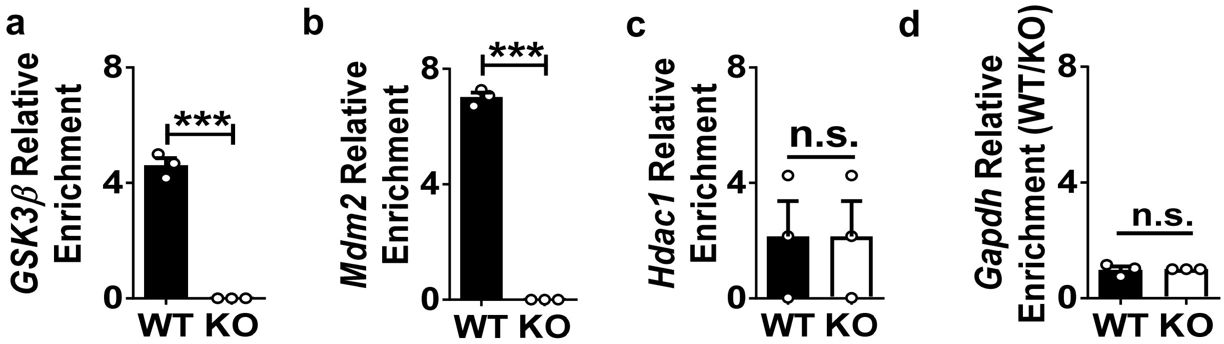

Supplementary Figure 4: FMRP RNA immunoprecipitation (IP) followed by quantitative real-time PCR analyses for *GSK3β* , *Mdm2, Hdac1* and *Gapdh* mRNAs in WT and *Fmr1* KO mouse NPCs.

FMRP was associated with *GSK3β* (**a**, positive control) and *Mdm2* (**b**), but not *Hdac1* (**c**) or *Gapdh* (**d**, negative control) mRNAs (n=3). ***P<0.001, n.s., no significant difference for relative fold enrichment when comparison of WT to *Fmr1* KO NPCs . Student's *t*-tests were used for data analyses. Data are presented as mean ± s.e.m.

For a-c (and **Figure 2h**), the enrichment of each gene was normalized to *Gapdh* and calculated as: 2^{-[(CtGskIP-CtGapdhIP)-(CtGskInput-CtGapdhInput)]}

For d, the enrichment of *Gapdh* in WT was normalized to KO and calculated as: 2^{-[(CtGapdhIPWT-CtGapdhInputWT)-(CtGapdhIPKO-CtGapdhInputKO)]}. KO was therefore set is 1 without error bars.

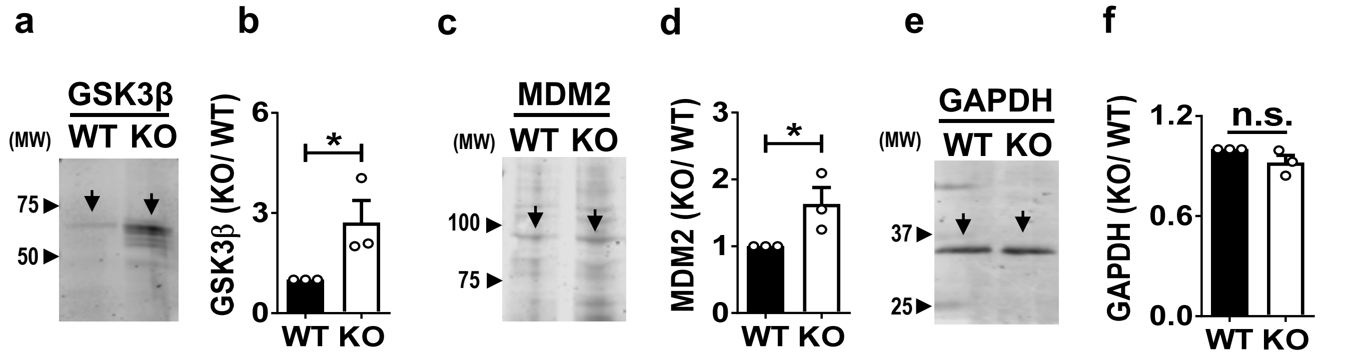

Supplementary Figure 5: **BONCAT (biorthogonal non-canonical amino acid tagging) followed by western blot analyses for GSK3β, and GAPDH protein in WT and *Fmr1* KO mouse NPCs.**

(**a, b**) Sample Western blot (**a**) and quantitative analysis (**b**, n=3) of newly-synthesized GSK3β protein in WT and *Fmr1* KO NPCs by using BONCAT. (**c, d)** Sample Western blot (**c**) and quantitative analysis (**d**, n=3) of newly-synthesized MDM2 protein in WT and *Fmr1* KO NPCs by using BONCAT. (**e, f**) Sample Western blots (**e**) and quantitative analyses (**f,** n=3) for detection of newly-synthesized GAPDH protein in WT and *Fmr1* KO NPCs using BONCAT, (n=3). *P<0.05, n.s., no significant difference. Student's *t*-tests were used for data analyses. Data are presented as mean ± s.e.m.

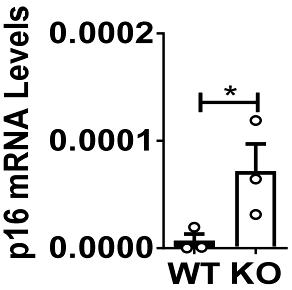

Supplementary Figure 6: *p16* mRNA expression levels in WT and *Fmr1* KO mouse NPCs

Quantitative real-time PCR analyses showing up-regulation of *p16* mRNA levels in *Fmr1* KO NPCs compared to WT controls, (n=3). *Gapdh* was used as an internal control. *P<0.05. Student's *t*-tests were used for data analyses. Data are presented as mean ± s.e.m.

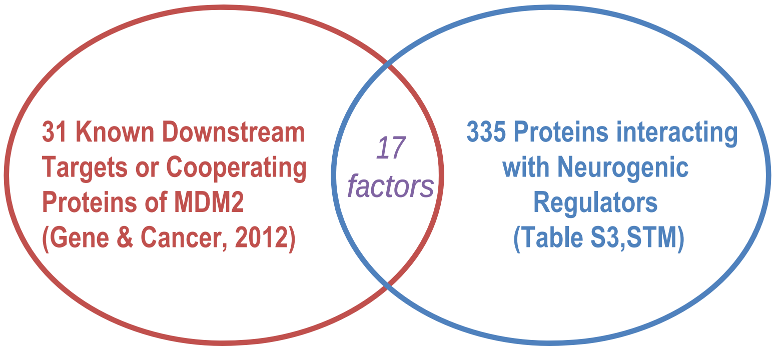

Factors	Downstream MDM2 targets?	MDM2 Cooperating Proteins?	Neurogenic Interactor?	# of neurogenic Interactions	# of supporting literatures
EP300		√	√	3	4
NUMB			√	3	4
XIAP		√	√	3	4
CBP		√	√	3	3
HDAC1	√		√	2	4
TP53	√		√	1	1
E2F1		√	√	1	N/A (GeneMania)
TAF1		√	√	1	N/A (GeneMania)
TBP		√	√	1	N/A (GeneMania)
AR	√		√	1	N/A (GeneMania)
HIPK2	√		√	1	1
IGF1R	√		√	1	1
NICD4 (NOTCH4)	√		√	1	1
RPS27L	√		√	1	N/A (GeneMania)
Nbs1 (NBN)		√	√	1	1
CHK2 (CHEK2)	√		√	1	N/A (GeneMania)
TP73	√			0	N/A (GeneMania)

Supplementary Figure 7: Evaluation of candidate proteins that may interact with MDM2 to regulate neurogenesis. ***Upper***: Workflow for selecting potential MDM2 targets that may regulate activation and maintenance of NSCs. ***Lower:*** Columns in red indicate evidence that a given factor is either a downstream MDM2 target or is known to physically cooperate with MDM2. The blue column indicates evidence that a given factor is known to physically interact with a set of 16 well-established "Neurogenic Regulators" curated from the MANGO database and literature search. The absence of a check mark for TP73 indicates that TP73 is itself among the 16 Neurogenic Regulators. The column entitled "# of neurogenic interactions" indicates the number of Neurogenic Regulators with which a given factor is known to have physical interactions, and is used to reflect the likelihood that a candidate factor may have a role in neurogenesis. The column entitled "# of supporting literatures" indicates the number of publications providing evidence for the physical interaction between a candidate factor and Neurogenic Regulators as given by BioGrid 3.4. In this column, "N/A (GeneMania)" indicates that evidence of the physical interaction was curated by GeneMania, which does not report the number of publications.

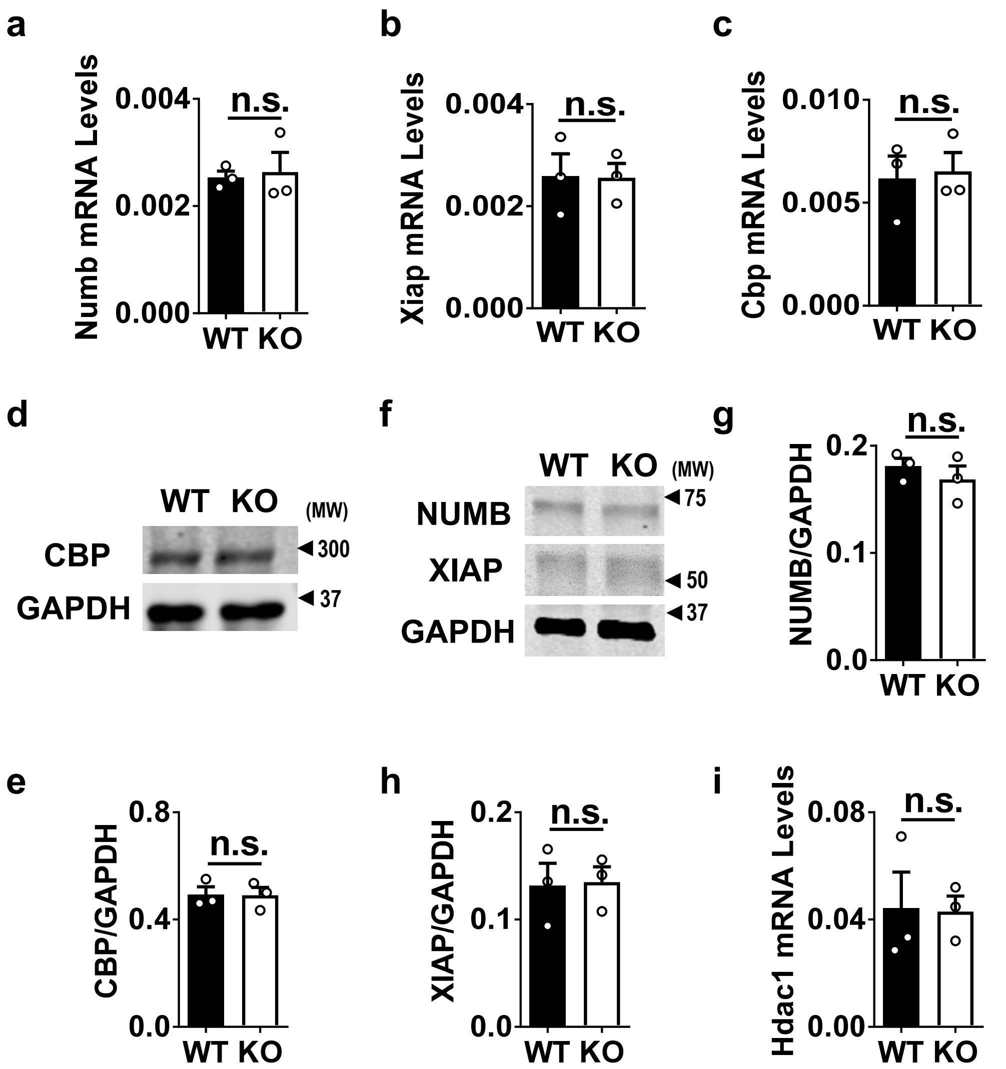

Supplementary Figure 8: **Differential changes of candidate MDM2 effectors in NPCs.** (**a, b, c**) Quantitative real-time PCR analyses showing no significant differences between WT and *Fmr1* KO 6-mo NPCs in *Numb* (**a**), *Xiap* (**b**), and *Cbp* (**c**) mRNA levels, (n=3). *Gapdh* was used as an internal control. (**d, e, f, g, h**) Western blot analyses showing no significant difference between WT and *Fmr1* KO NPCs in CBP protein (**d**, **e**), NUMB protein (**f**, **g**) and XIAP protein (**f**, **h**) levels, (*n* = 3). GAPDH was used as a loading control. (**i**) Quantitative real-time PCR analyses showing no significant differences between WT and *Fmr1* KO 6-mo NPCs in *Hdac1* mRNA level, (n=3). *Gapdh* was used as an internal control. Student's *t*-tests were used for data analyses. Data are presented as mean ± s.e.m.

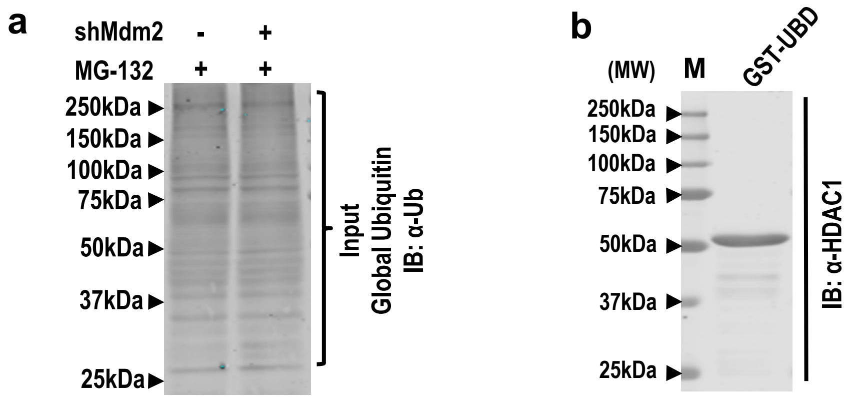

Supplementary Figure 9: Global ubiquitin expression did not change in NPCs with MDM2 knockdown (+shMdm2) and control (-shMdm2).

(**a**) Western blot analyses of ubiquitin expression in NPCs with shMdm2 or control treatment in the presence of a proteasome inhibitor (MG-132). (**b**) Western blot analyses of HDAC1 in GST-UBD alone (without sample lysate) to identify the reaction of GST-UBD and HDAC1 antibody may only form the band at around 50kDa. M: Marker.

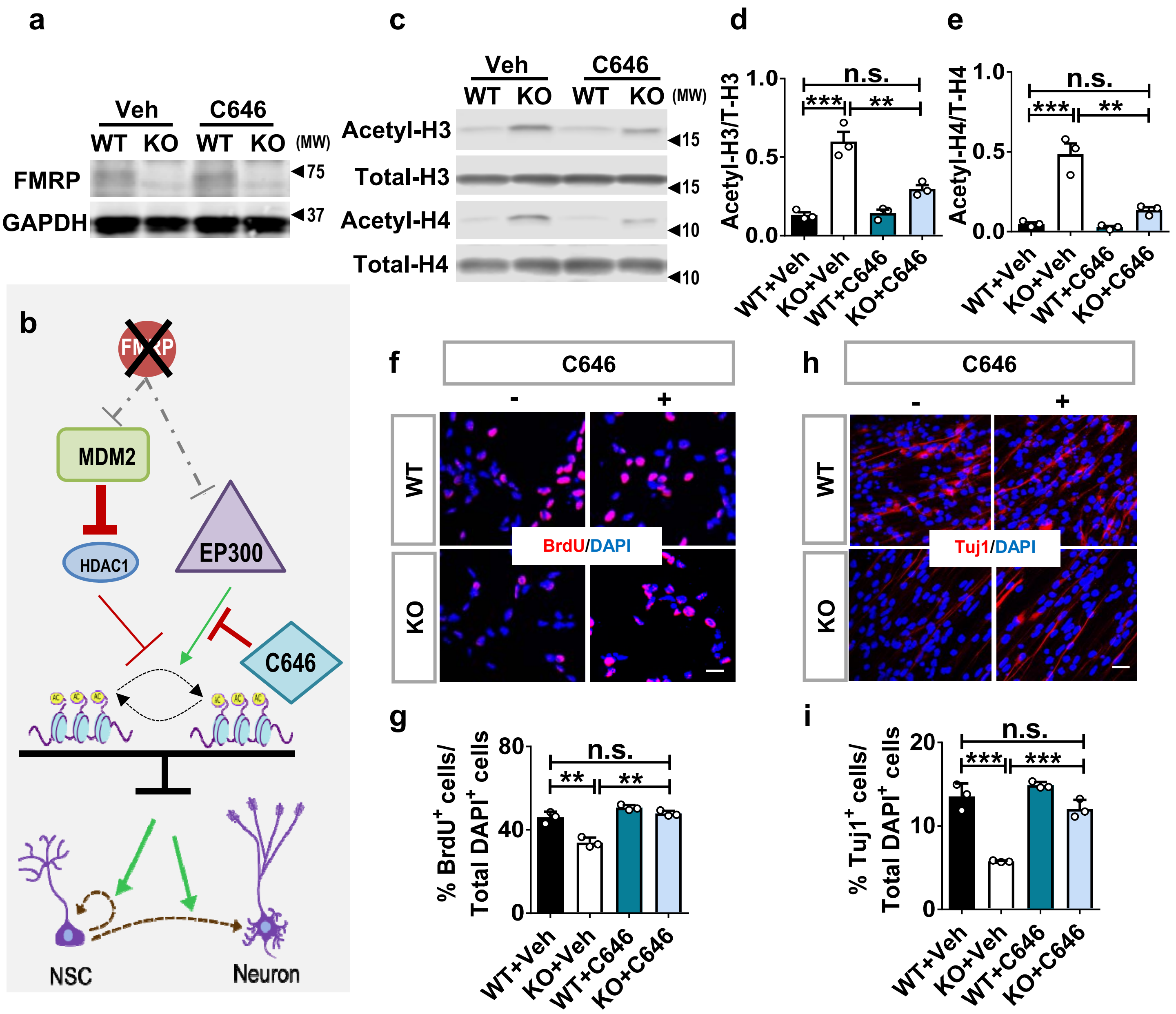

Supplementary Figure 10: **Rebalancing histone acetylation induced by EP300 inhibitor C646 rescues proliferation and differentiation of *Fmr1* KO 6-mo NPCs.**
(a) FMRP protein expression in primary *Fmr1* KO and WT NPCs treated with vehicle or C646.(**b**) Schematic model showing that C646 treatment rebalances histone acetylation and rescues proliferation and differentiation of *Fmr1* KO NPCs. (**c-e**) Western blot analyses (**c**) of acetylated and total histone H3 (**d)** and histone H4 (**e**) in NPCs with C646 treatment, (n = 3). Total histone H3 and total histone H4 were used as a loading control for acetylation of histone H3 and acetylation of histone H4, respectively. (**f, g**) C646 treatment rescued the cell proliferation phenotype of *Fmr1* KO NPCs, as assessed by BrdU incorporation (**e**, Red, BrdU; Blue, DAPI. Scale bar, 20μm) followed by quantitative analysis of BrdU^{+} cells (**f**), (n = 3). (**h, i**) C646 treatment rescued neuronal differentiation phenotypes of *Fmr1* KO NPCs as assessed by a neuronal marker Tuj1^{+} (**g**, Red, Tuj1; Blue, DAPI. Scale bar, 20μm) followed by quantitative analysis of Tuj1^{+} cells (**h**), (n = 3). **P<0.01, ***P<0.001, n.s., no significant difference. Two-Way ANOVA was used for data analyses in d, e, g and i. Data are presented as mean ± s.e.m.

a

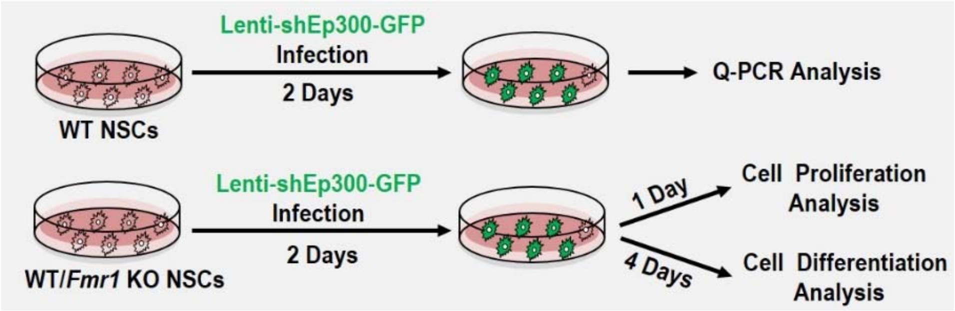

b

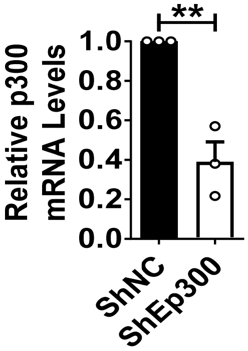

Supplementary Figure 11: Acute knockdown of EP300 in NPCs
(**a**) Experimental scheme for EP300 acute knockdown experiment. WT NPCs were infected with lentivirus expressing either *shEp300* (Lenti-*shEp300*-GFP) or control s*hNC* (Lenti-*shNC*-GFP). (**b**) Quantitative real-time PCR analyses showing Lenti-*shEp30*-GFP infected cells had significantly decreased *Ep300* mRNA levels compared to Lenti-*shNC*-GFP infected cells (n=3). **P<0.01. Student's t-tests were used for data analyses. Data are presented as mean ± s.e.m.

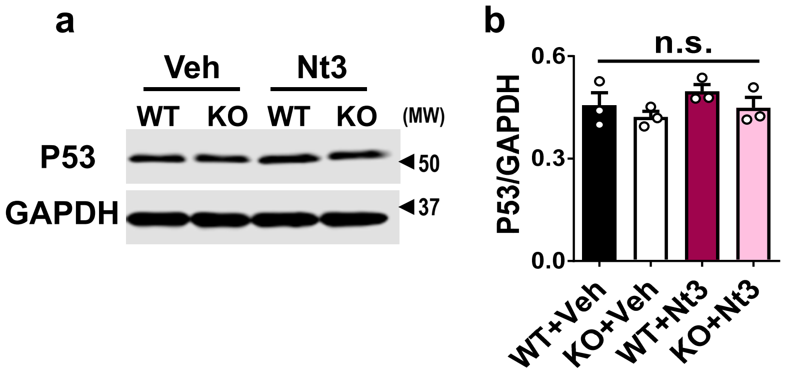

Supplementary Figure 12: Nutlin-3 treatment does not affect P53 levels in *Fmr1* KO 6-mo NPCs. (**a, b**) Western blot analyses of P53 protein expression in WT and *Fmr1* KO 6-mo NPCs (n = 3). n.s., no significant difference versus all other groups. Two-Way ANOVA was used for data analyses. Data are presented as mean ± s.e.m.

a

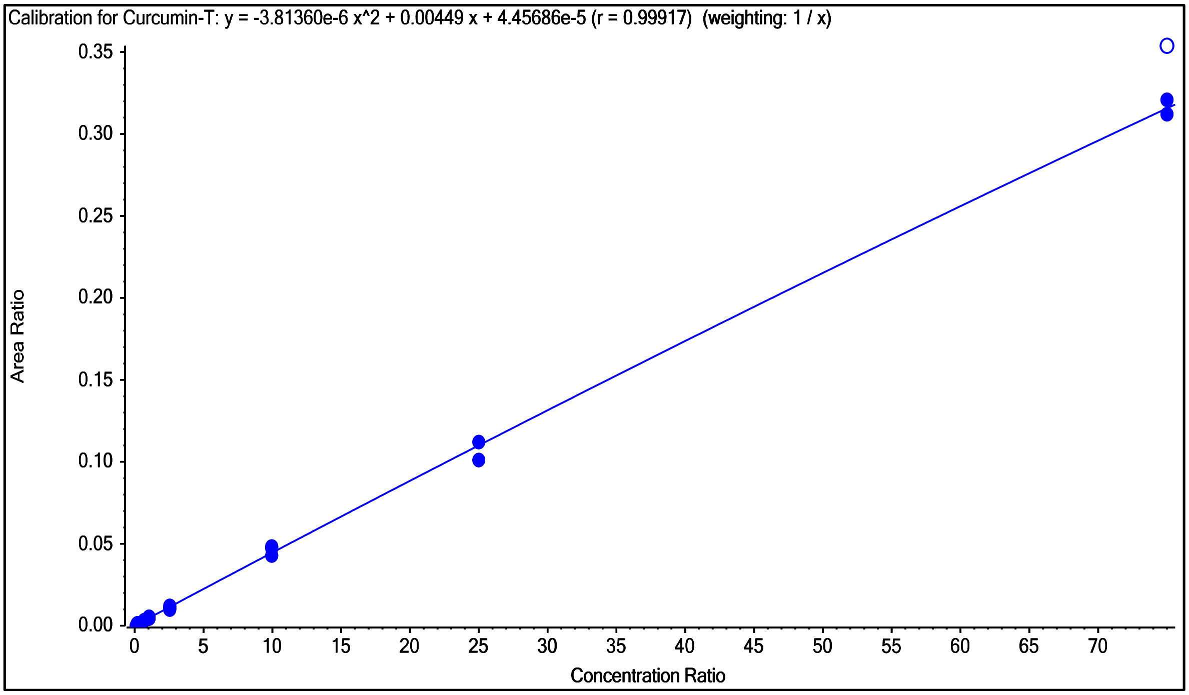

b

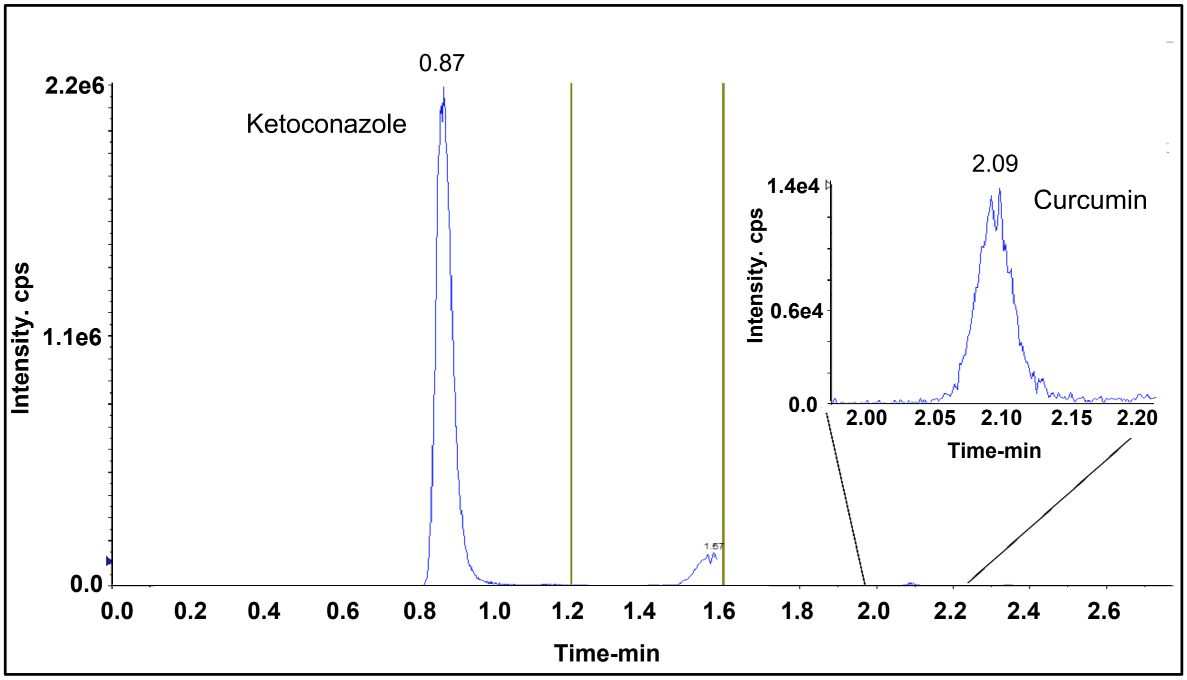

c

Mouse	Average Concentration (ng mL^{-1})	Average Concentration (ng g^{-1} tissue)
20180314_C1	0.106	2.12
20180314_C2	0.052	1.04
20180314_C3	0.160	3.20
**Average**	**0.106**	**2.12**

Supplementary Figure 13: Curcumin concentration in mouse brains analyzed at 60 min after a single i.p. injection (0.4 mg kg^{-1}).
(**a**) Standard curve of Curcumin between 0.1 and 75 ng mL^{-1}. (r=0.99917).
(**b**) Curcumin peak detected by LC-MS-MS. Ketoconazole is the internal control detected by LC-MS-MS
(**c**) Concentration of curcumin measured in three mouse brains (three animals: C1, C2, C3).

a

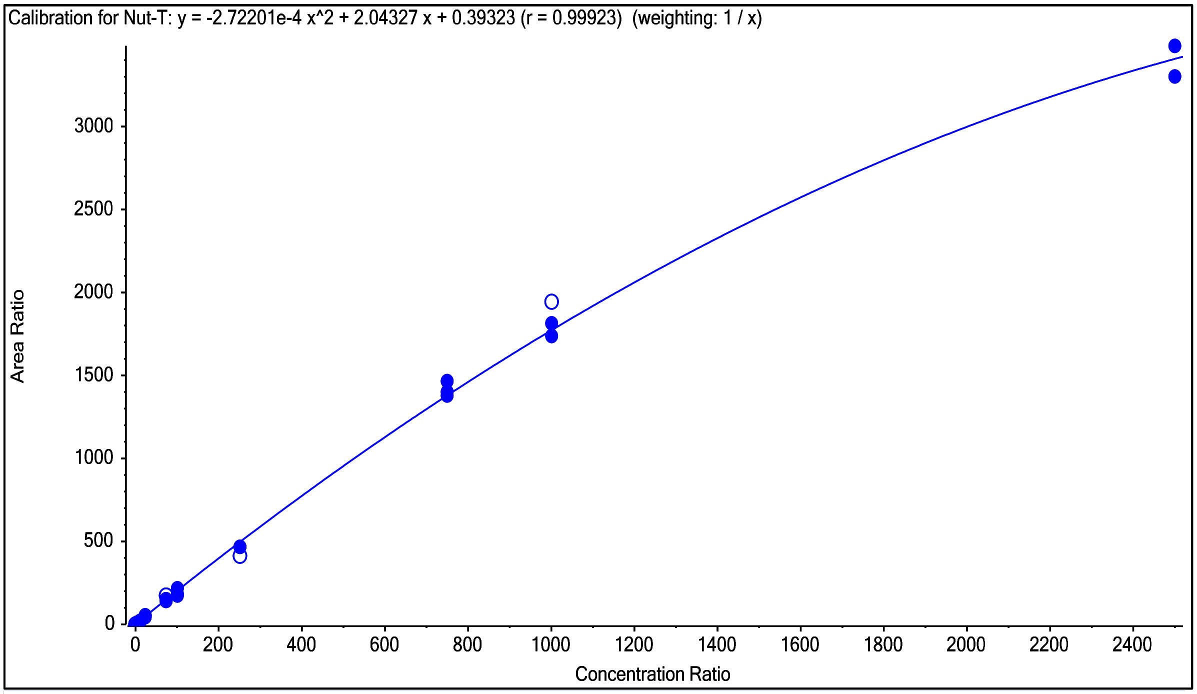

b

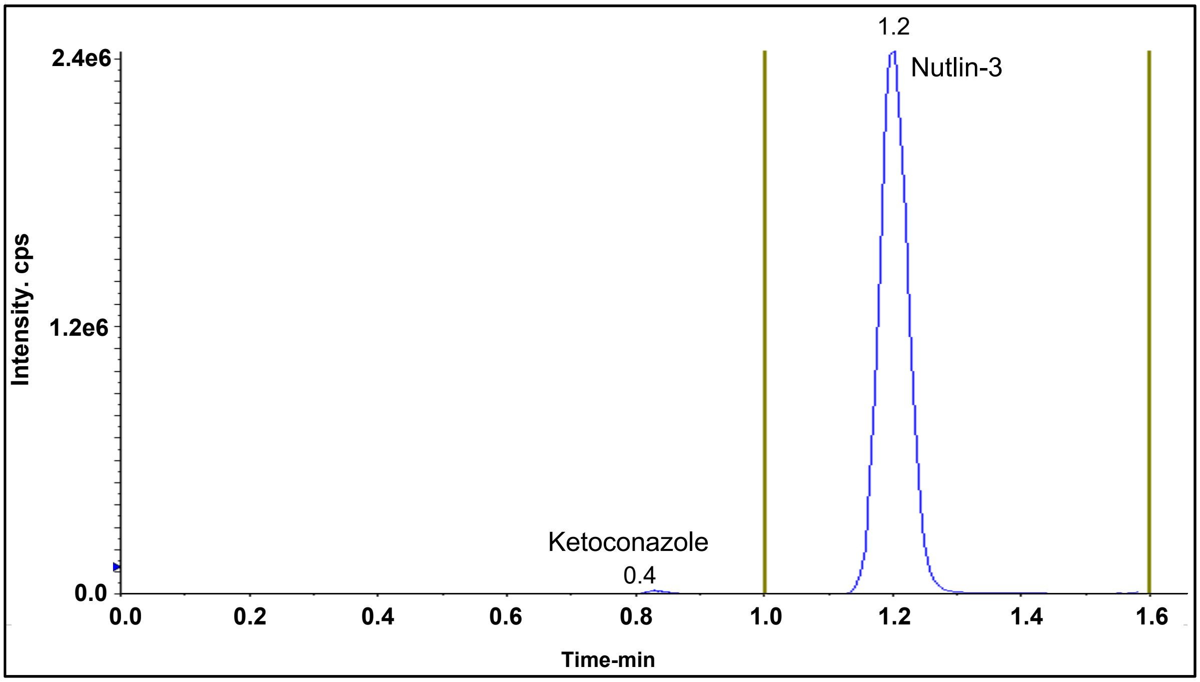

c

Mouse	Average Concentration (ng mL^{-1})	Average Concentration (ng g^{-1} tissue)
20180307_N2	33.65	1346.07
20180307_N3	40.05	1602.02
20180307_N4	23.58	943.00
**Average**	**32.43**	**1297.03**

Supplementary Figure 14:. Nutlin-3 concentration in mouse brains analyzed at 60 min after a single i.p. injection (8 mg kg^{-1}).
(**a**) Standard curve of Nutlin3 between 1 and 2500 ng mL^{-1}. (r=0.99923).
(**b**) Nutlin-3 peak detected by LC-MS-MS. Ketoconazole is the internal control detected by LC-MS-MS.
(**c**) Concentration of Nutlin3 measured in three mouse brains (three animals: N2, N3, N4).

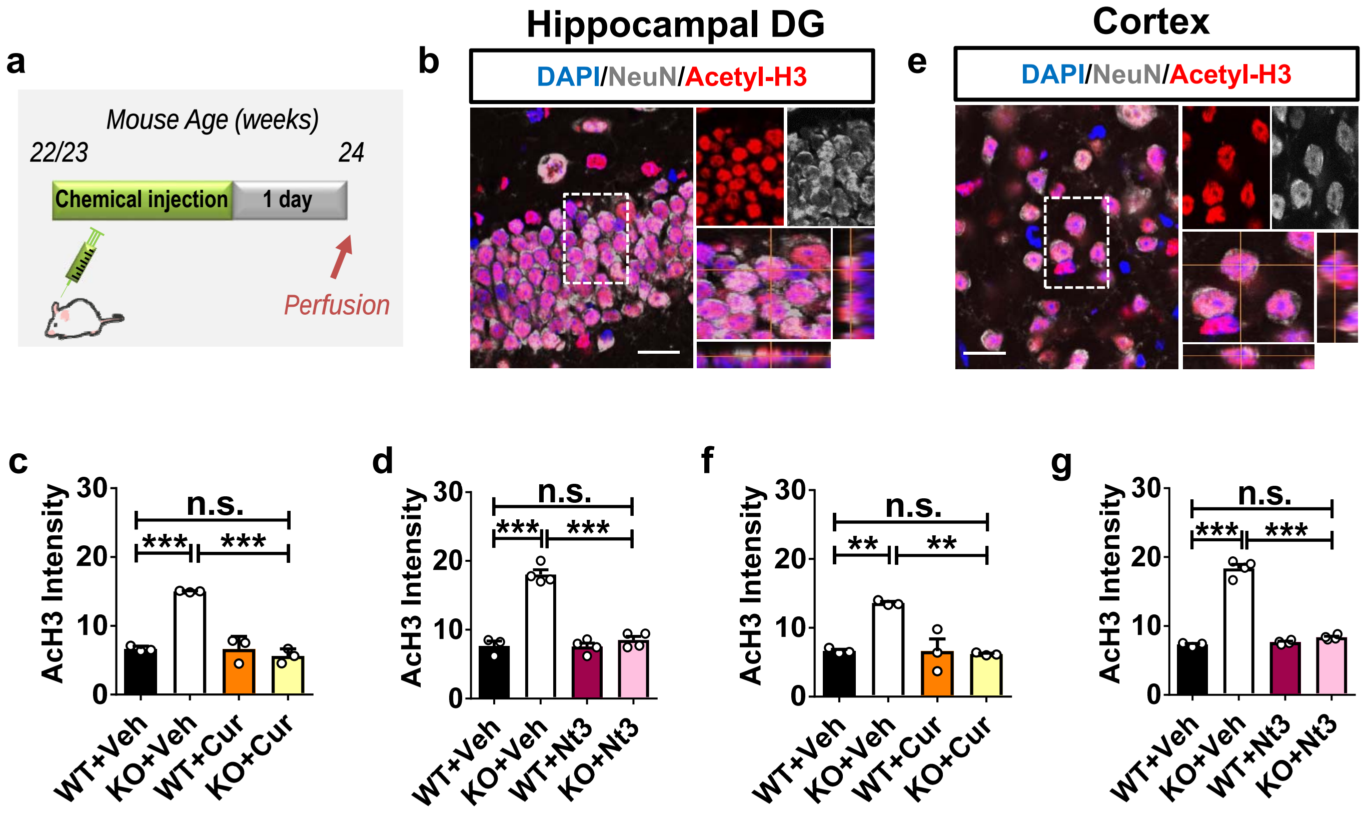

Supplementary Figure 15: Histone acetylation levels are elevated in NeuN+ neurons of hippocampal DG and cortex in *Fmr1* KO mice.

(**a**) Experimental schemes for assessing histone acetylation in WT and *Fmr1* KO mice treated with vehicle, curcumin or Nutlin-3. (**b**) Sample confocal images of acetylated histone H3 (Acetyl-H3) in the hippocampal DG of 6-month old WT and *Fmr1* KO mice. Blue, DAPI; Grey, NeuN; Red, Acetyl-H3. Scale bar, 20μm. (**c, d**) curcumin treatment (**c**, *n*=3 mice per group) and Nutlin-3 (**d**, *n*=3 to 4 mice per group) reduced Histone H3 acetylation in NeuN+ neurons in the DG of *Fmr1* KO mice without affecting WT mice. (**e**) Sample confocal images of acetylated histone H3 in layerII/III of the somatosensory cortex in 6-month old WT and *Fmr1* KO mice. Blue, DAPI; Grey, NeuN; Red, Acetyl-H3. Scale bar, 20μm. (**f, g**) curcumin treatment (**f**, *n*=3 mice per group) and Nutlin-3 (**g**, *n*=3 to 4 mice per group) reduced Histone H3 acetylation in NeuN+ neurons in *Fmr1* KO cortex without affecting WT mice.**P<0.01, ***P<0.001, n.s., no significant difference. Two-way ANOVA was used to all data analyses. Data are presented as mean±s.e.m. The boxes with dotted white lines in (**b**) and (**e**) indicate regions where high magnification images are shown.

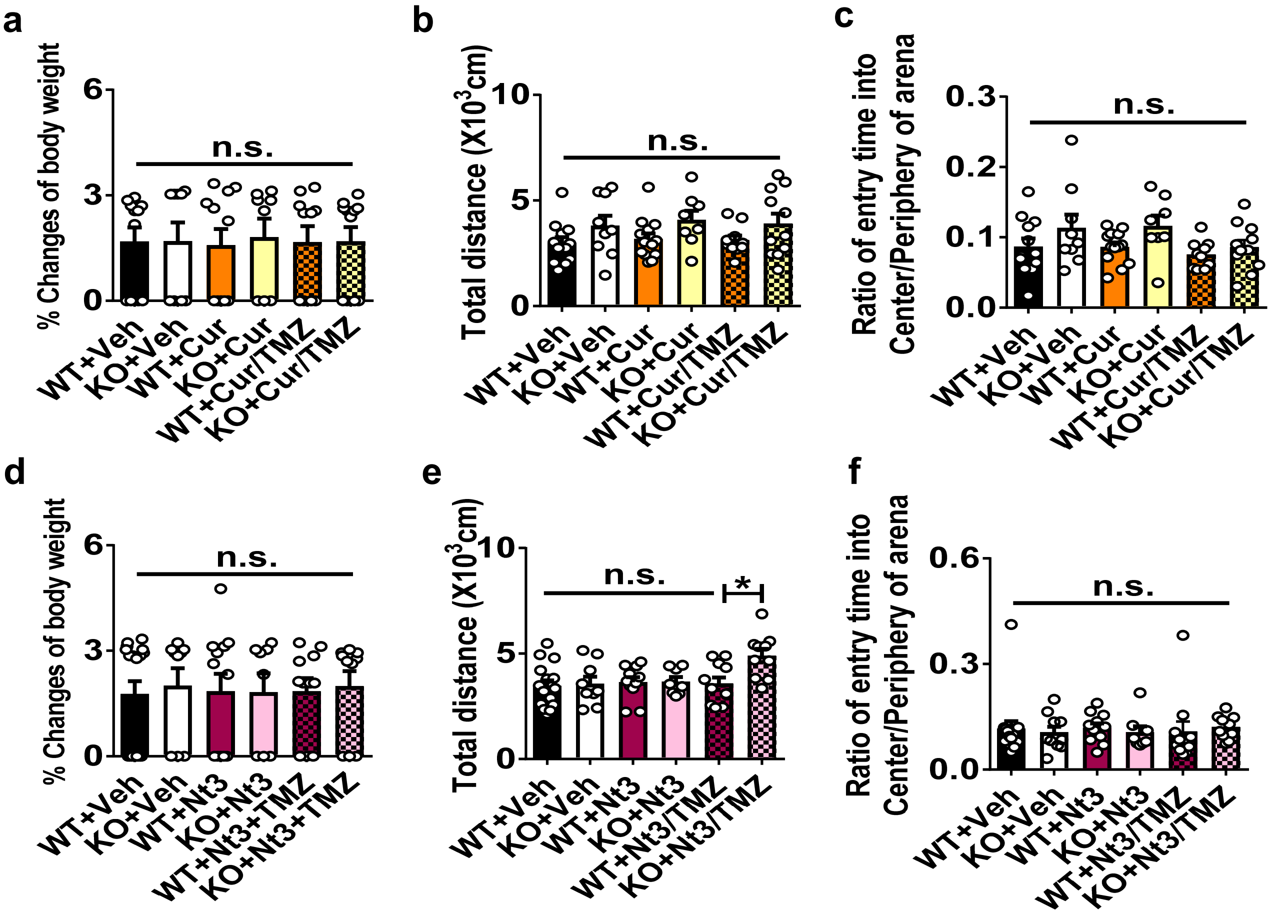

Supplementary Figure 16: **Concurrent TMZ and chemical inhibitor treatment does not affect general health and activities of WT and *Fmr1* KO mice.**

(**a, b, c**) Concurrent TMZ treatment does not affect body weight (**a**), (*n*=8-11 mice per group) or locomotor activities, including total movement distances (**b**), (*n*=8-13 mice per group) and the ratio of number of center over periphery entries (**c**), (*n*=8-13 mice per group) in WT and *Fmr1* KO mice treated with curcumin. (**d, e, f**) Concurrent TMZ treatment does not affect body weight (**d**), (*n*=8-17 mice per group) or locomotor activities and anxiety, including total movement distances (**e**), (*n*=8-15 mice per group), and the ratio of center over periphery entry (**f**), (*n*=8-15 mice per group), in WT and *Fmr1* KO mice treated with Nutlin-3. *$P<0.05$. n.s., no significant difference. Two-way ANOVA was used to all data analyses. Data are presented as mean ± s.e.m.

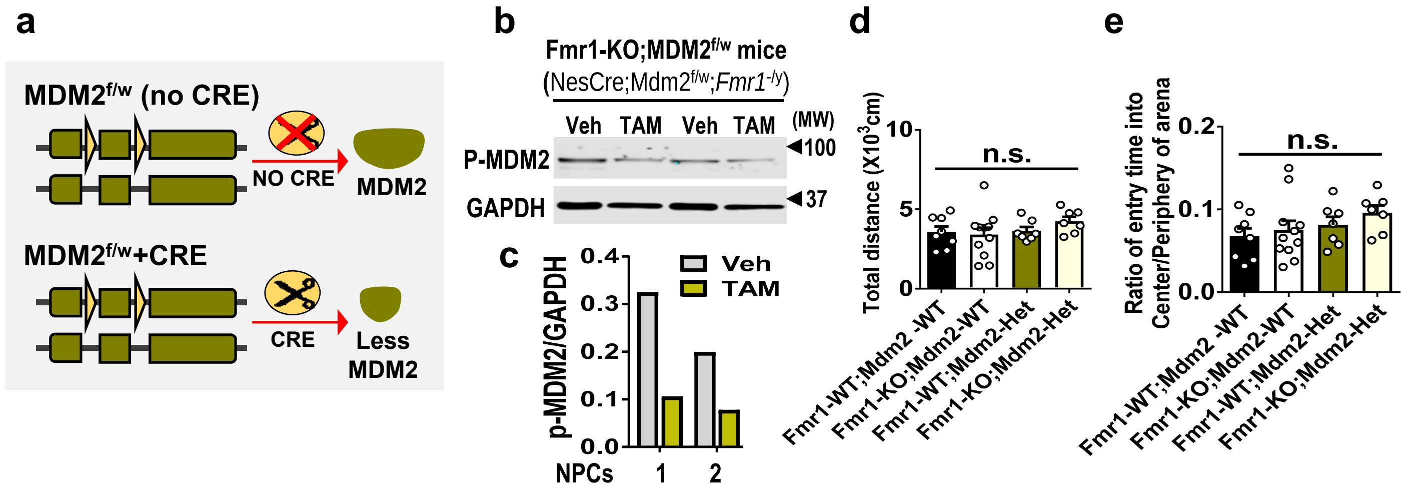

Supplementary Figure 17: **Heterozygote deletion of MDM2 in Nestin-expressing adult NSCs and new neurons does not affect locomoter activities of WT and *Fmr1* KO mice**. (**a**) In MDM2 Heterogeneous floxed ($MDM2^{f/w}$) cells, the presence of Cre lead to deletion of Mdm2 gene in floxed allele and reduced MDM2 protein levels. (**b, c**) Addition of 4-hydroxyl tamoxifen (TAM), but not vehicle (Veh), to NPCs isolated from *Nestin-CreER^{T2};MDM2$^{f/w}$;Fmr1* KO mice (Fmr1-KO;Mdm2-Het) led to reduced P-MDM2 (phosphorylated-MDM2) protein levels (n=2 NPC lines isolated from two mice); GAPDH was used as a loading control. (**d, e**) Upon tamoxifen injection (see **Figure 9a**), heterozygote deletion of MDM2 in Nestin-expressing adult NSCs and new neurons does not affect locomotor activities and anxiety, including total movement distances (**d**) (*n*=7-11 mice per group) and the ratio of number of center over periphery entries (**e**) (n=7-11 mice per group) in WT and *Fmr1* KO mice. n.s., no significant difference. Two-way ANOVA was used to all data analyses. Data are presented as mean ± s.e.m.

The genotypes of the mice include the following:

Fmr1-WT;Mdm2-WT: *Mdm2$^{w/w}$;Fmr1$^{+/y}$* or *NesCre;Mdm2$^{w/w}$Fmr1$^{+/y}$ or Mdm2$^{f/w}$;Fmr1$^{+/y}$*

Fmr1-KO;Mdm2-WT: *Mdm2$^{w/w}$;Fmr1$^{-/y}$ or Mdm2$^{f/w}$;Fmr1$^{-/y}$* or *NesCre;Fmr1$^{-/y}$;Mdm2$^{w/w}$,*

Fmr1-WT;Mdm2-Het: *NesCre;Mdm2$^{f/w}$;Fmr1$^{+/y}$*

Fmr1-KO;Mdm2-Het: *NesCre;Mdm2$^{f/w}$Fmr1$^{-/y}$*

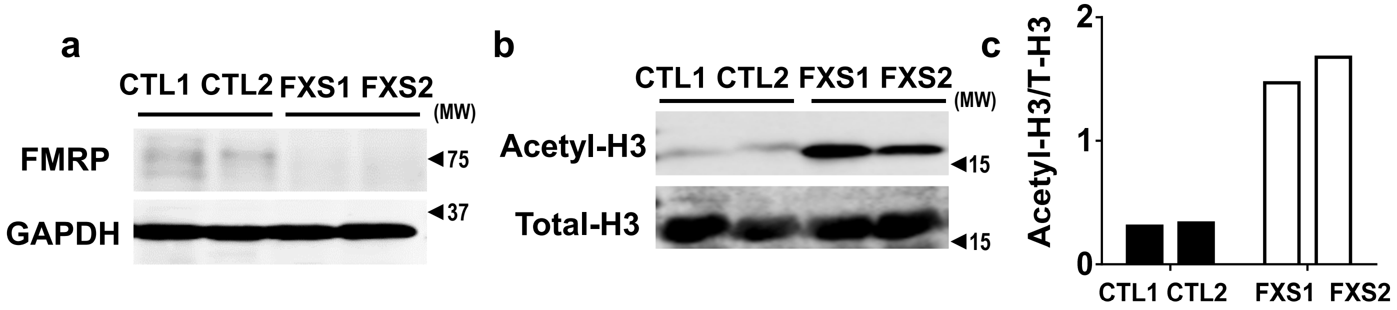

Supplementary Figure 18: Increased acetylation of histone H3 in the postmortem hippocampal tissue from FXS patients.

(a) Western blot analysis showing the absence of FMRP protein in the postmortem hippocampal tissue from two FXS patients (FXS1 and FXS2) .

(b-c) Western blot (**b**) and quantitative analyses of acetylated histone H3 (**c**) in the hippocampal tissues from two FXS patients (FXS1 and FXS2) and two matched control individuals (CTL1 and CTL2). Total Histone H3 was used as a loading control in western blot analysis.

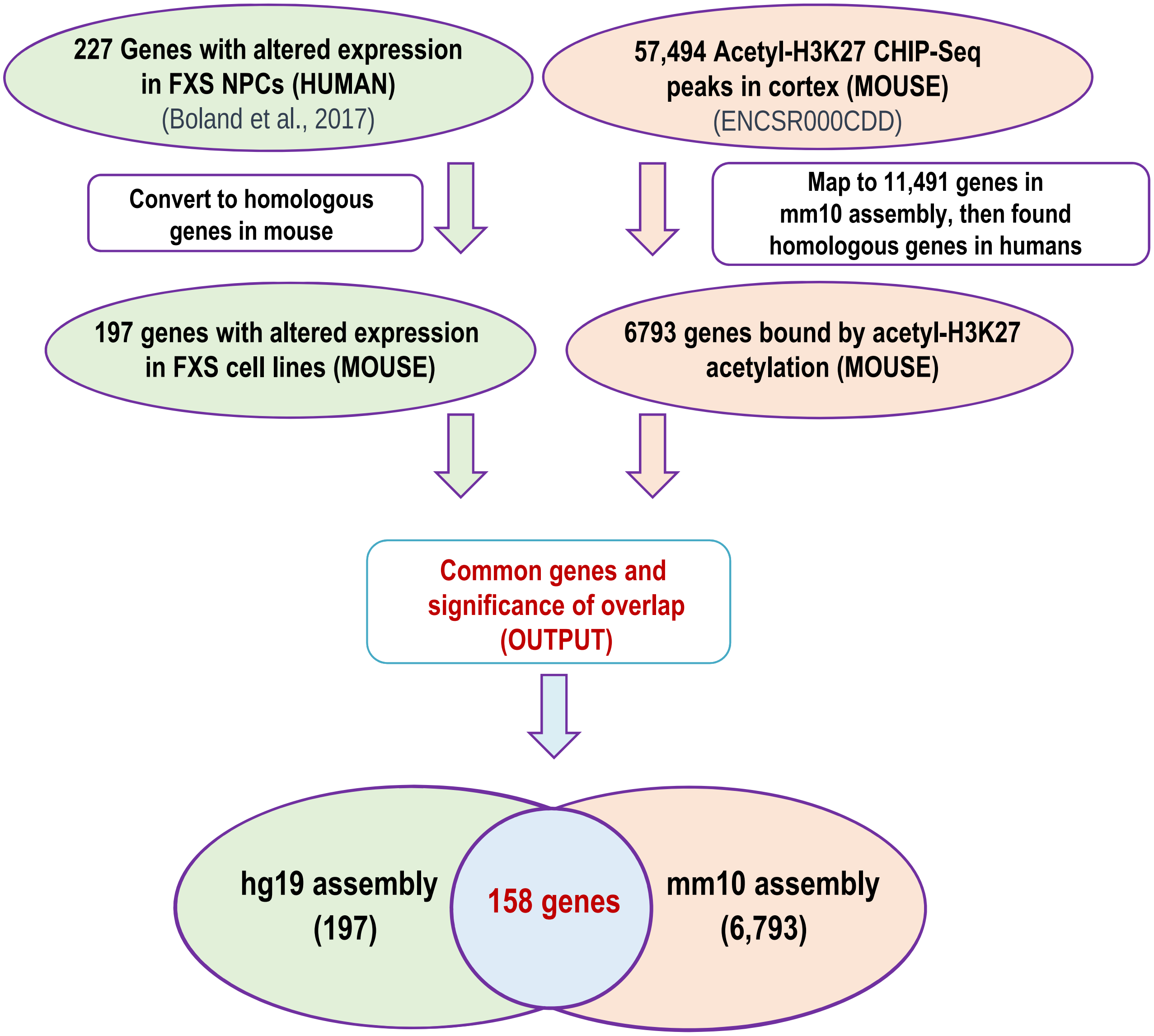

Supplementary Figure 19: Assessing correlation between transcriptomic changes in FMRP-deficient cells and genomic binding by acetylated histone H3.
The 227 genes with altered expression in NPCs differentiated from FXS patient-derived pluripotent stem cells FMRP-deficient human NPCs were obtained from literature (Boland et al 2017). Among them 197 genes have homologous counterparts in mice. Among acetyl-H3K27 bound mouse genes in the cortex, 6793 genes have human homologous genes. There is a significantly overlap between genes with altered expression in human FXS NPCs and genes with enriched acetyl-H3K27 binding in the mouse cortex (P=6.8e-9, 34% enrichment).

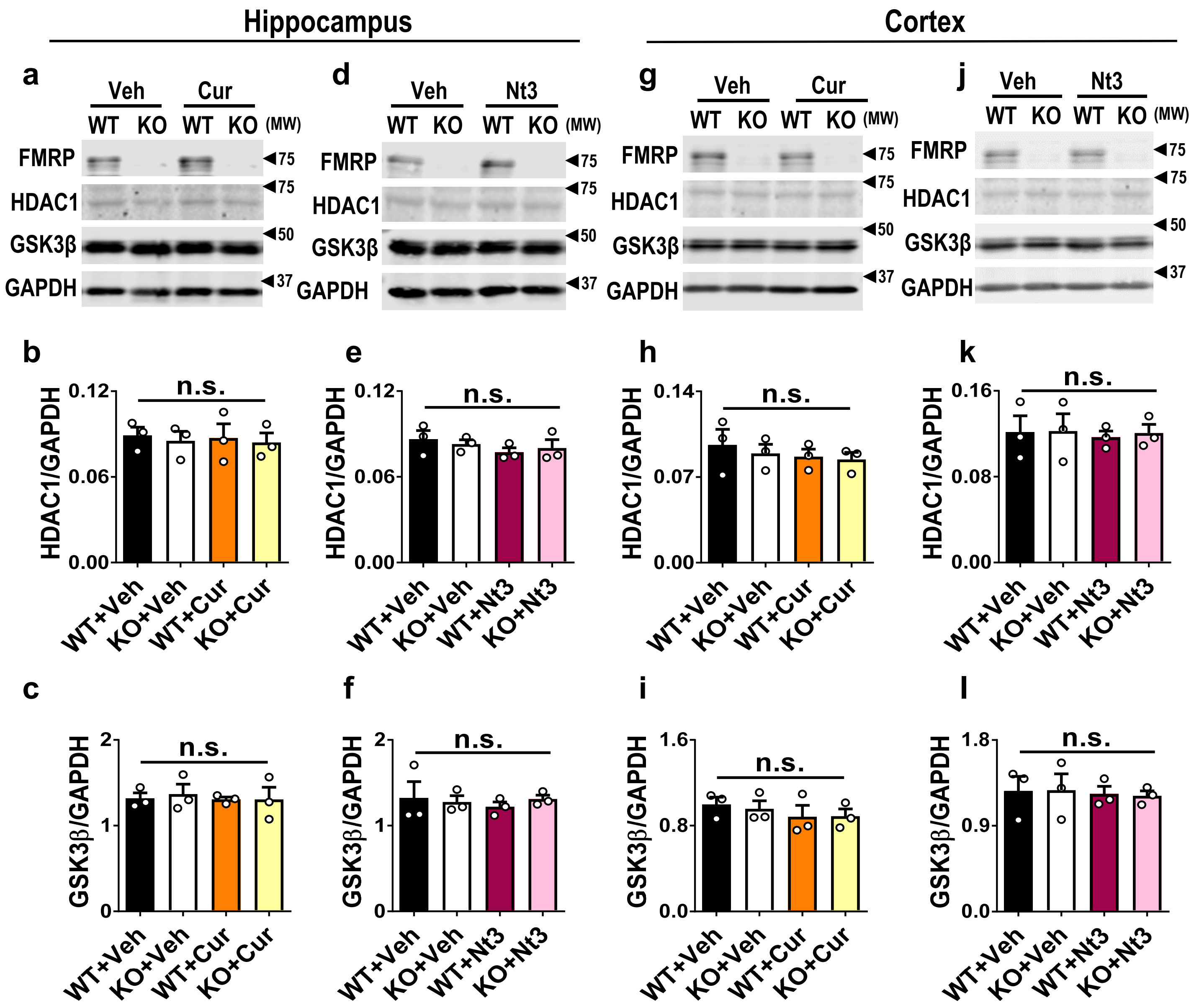

Supplementary Figure 20: Western blot analyses of HDAC1 and GSK3β protein levels in the hippocampus and cortex of 6-mo old WT and *Fmr1* KO mice treated with Vehicle, curcumin or Nutlin-3.

(a-c) HDAC1 and GSK3β protein levels in the hippocampus of WT and *Fmr1* KO mice treated with Vehicle (Veh) or Curcumin (Cur), (n=3). **(d-f)** HDAC1 and GSK3β protein levels in the hippocampus of WT and *Fmr1* KO mice treated with Vehicle (Veh) or Nutlin-3 (Nt3), (n=3). **(g-i)** HDAC1 and GSK3β protein levels in the cortex of WT and *Fmr1* KO mice treated with Vehicle (Veh) or Curcumin (Cur), (n=3). **(j-l)** HDAC1 and GSK3β protein levels in the cortex of WT and *Fmr1* KO mice treated with Vehicle (Veh) or Nutlin-3 (Nt3), (n=3). n.s., no significant difference. Two-Way ANOVA was used for data analyses. Data are presented as mean ± s.e.m. GAPDH was used as a loading control for all these experiments.

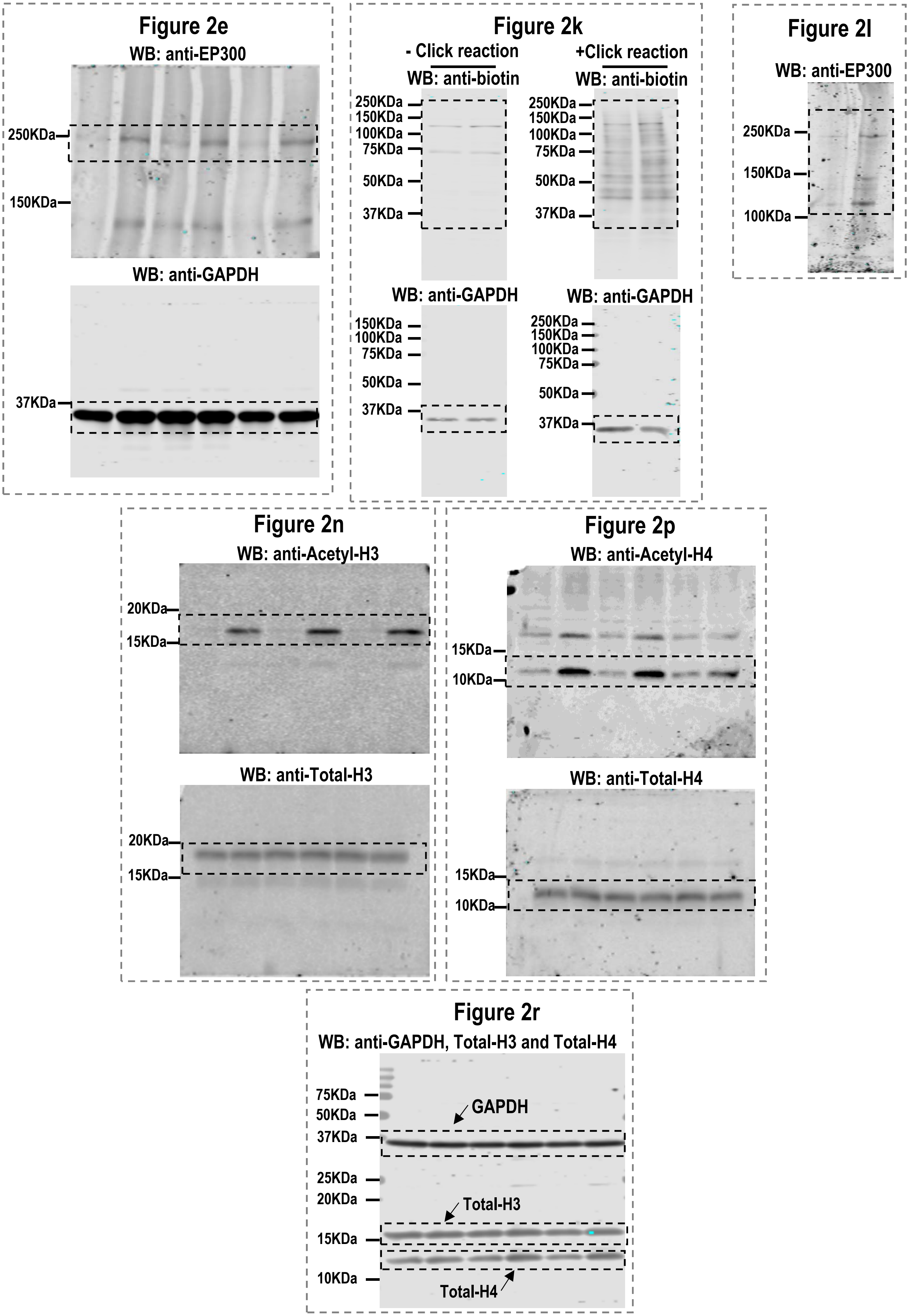

Supplementary Figure 21:The Full blot used for main figure 2

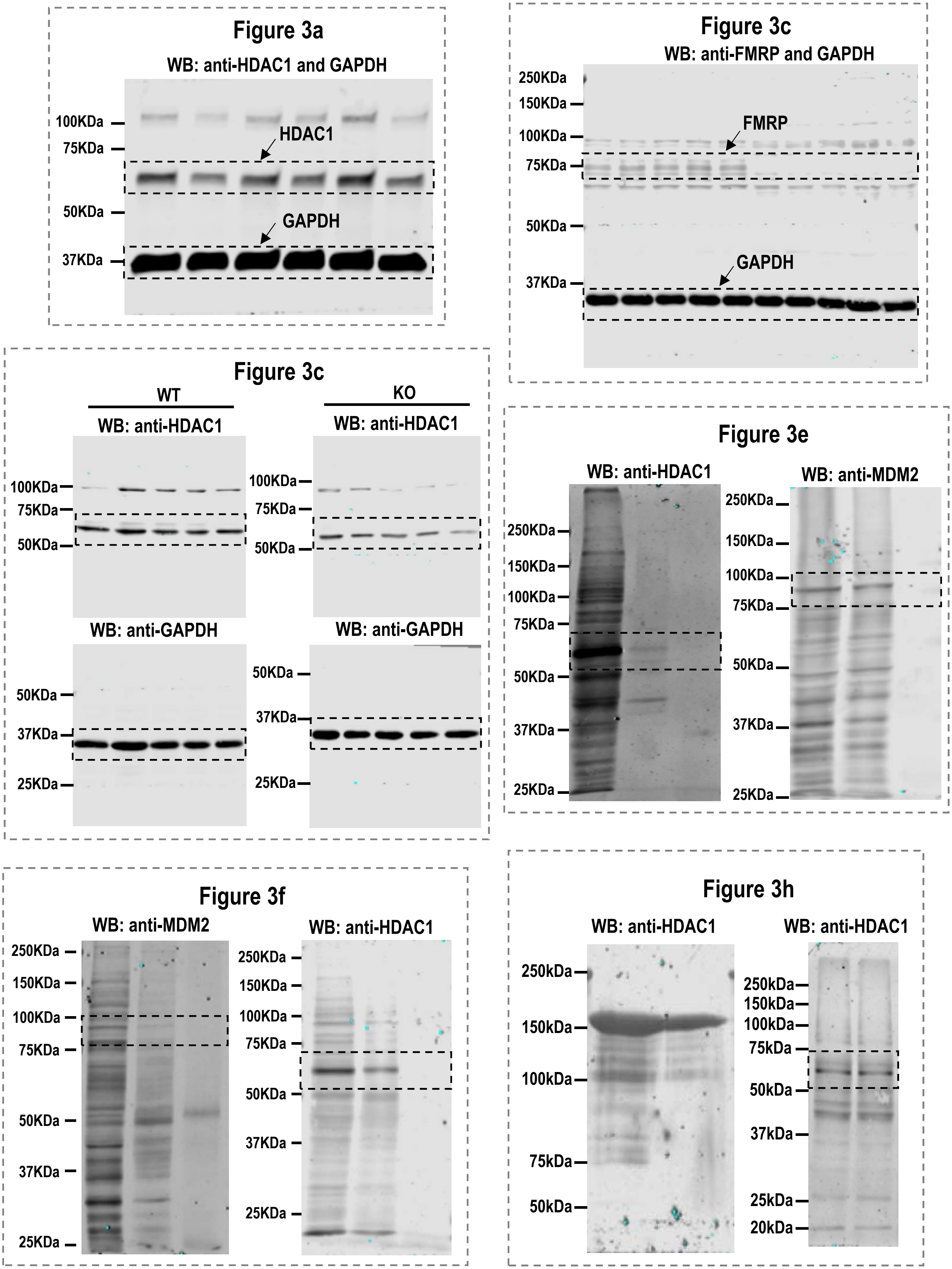

Supplementary Figure 22:The Full blot used for main figure 3

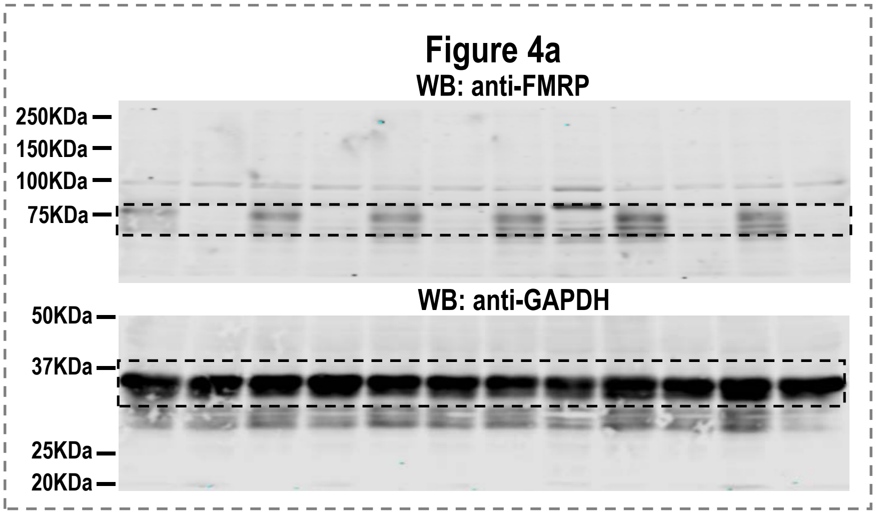

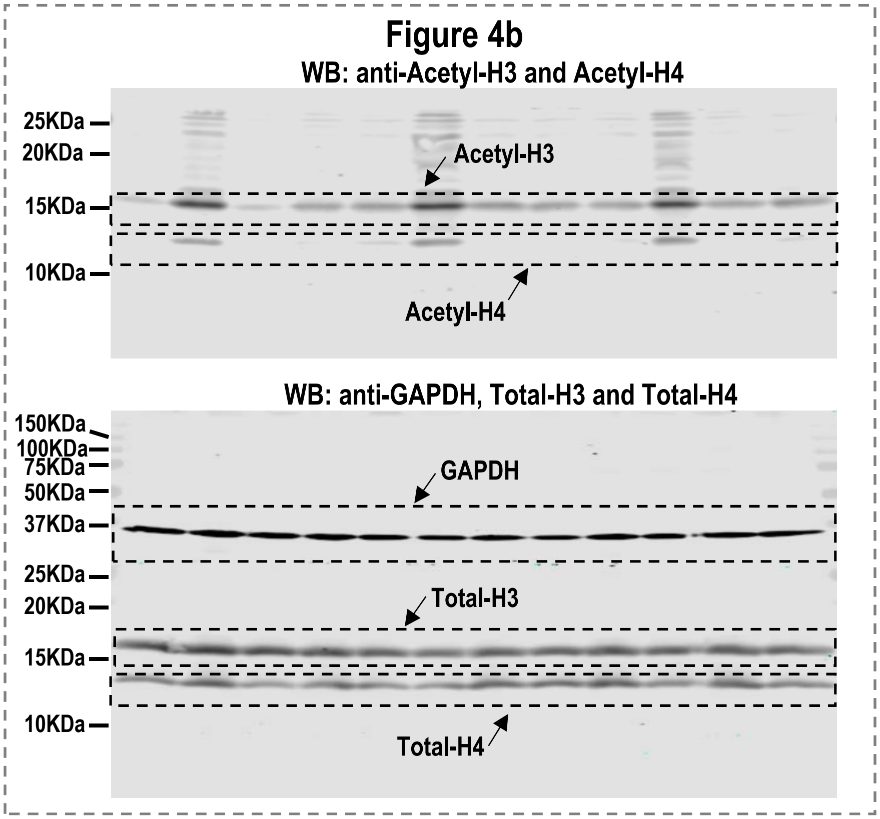

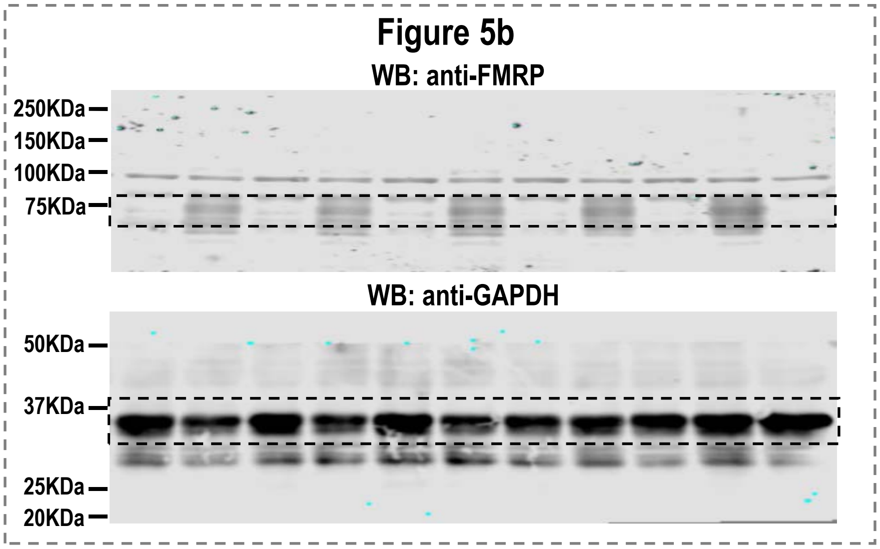

Supplementary Figure 23:The Full blot used for main figure 4 and figure 5b

Figure 5b

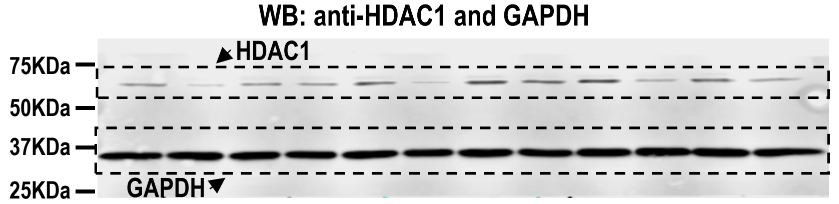

Figure 5d

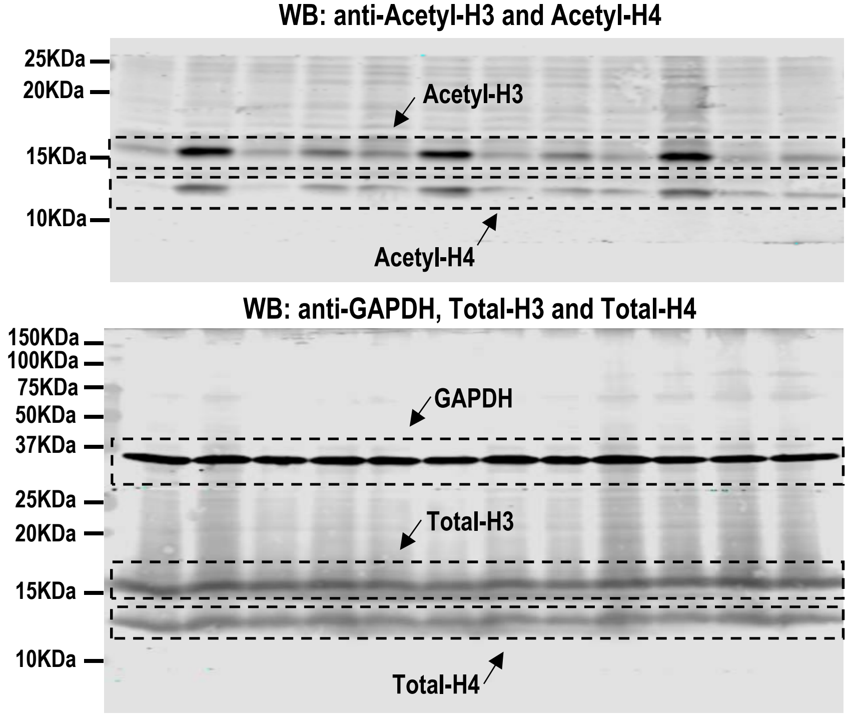

Supplementary Figure 3a

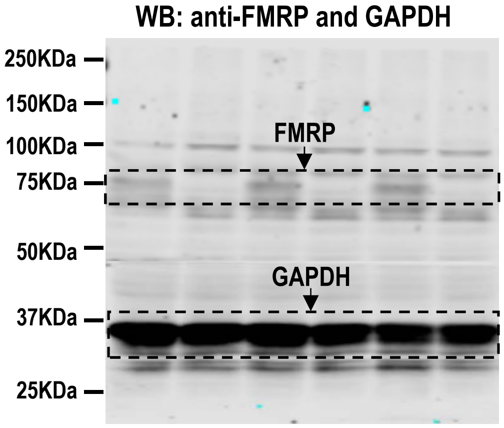

Supplementary Figure 3b

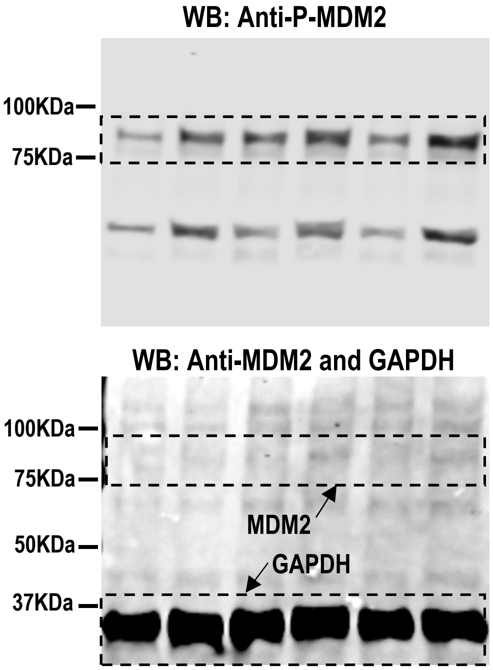

Supplementary Figure 3i

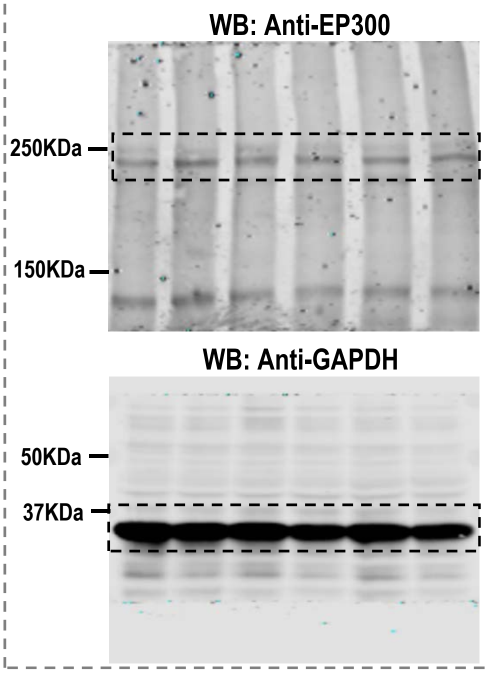

Supplementary Figure 3e

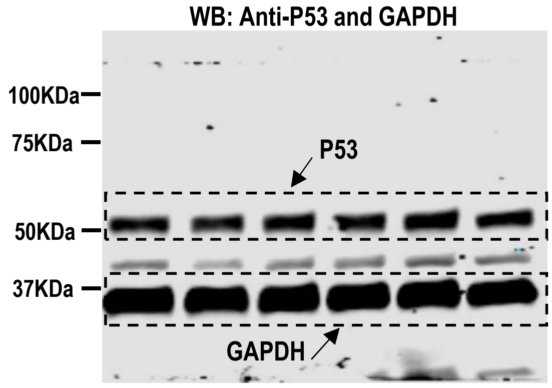

Supplementary Figure 24:The Full blot used for main figure 5b and supplementary figure 3

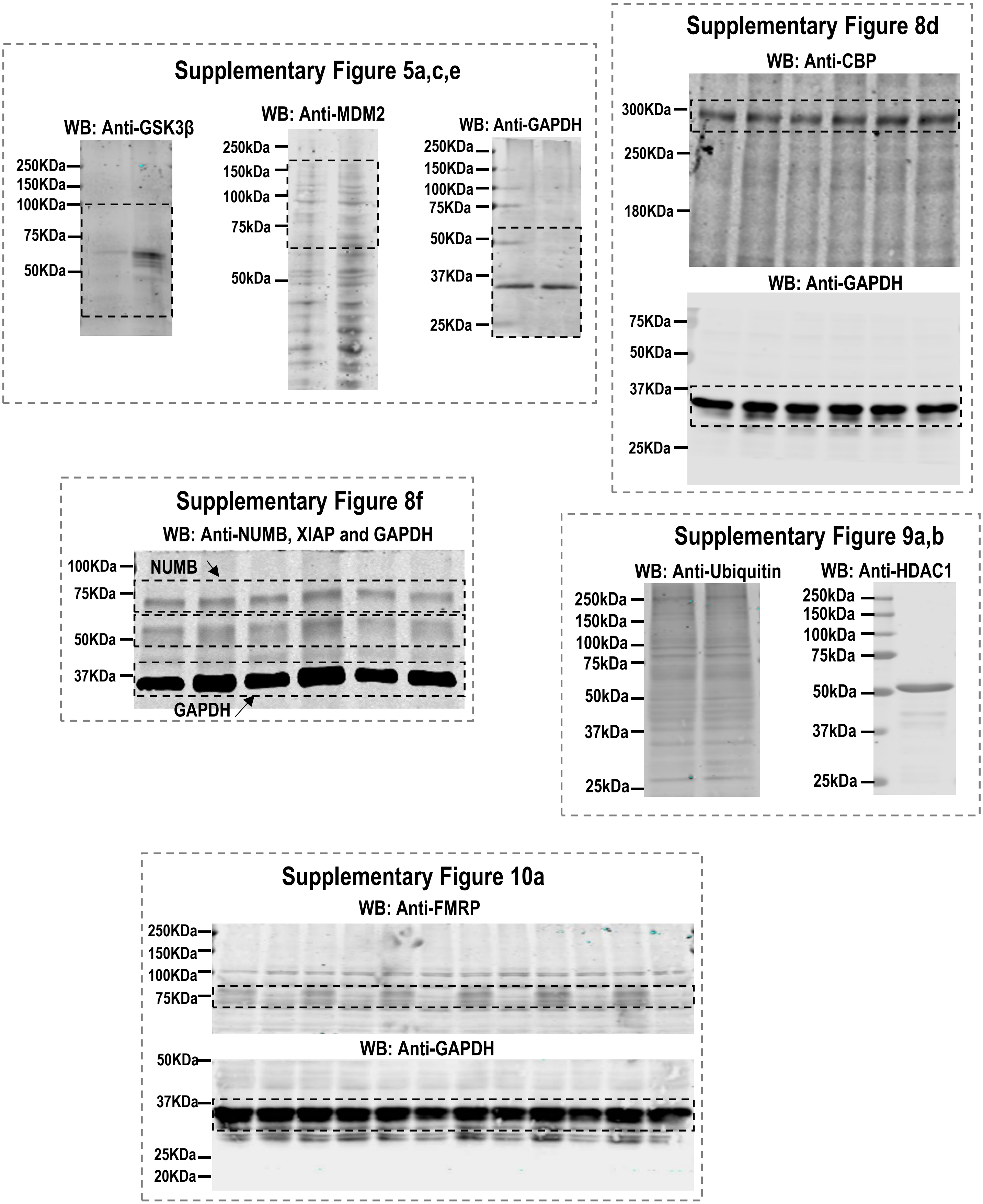

Supplementary Figure 25:The Full blot used for supplementary figures 5,8,9,10a

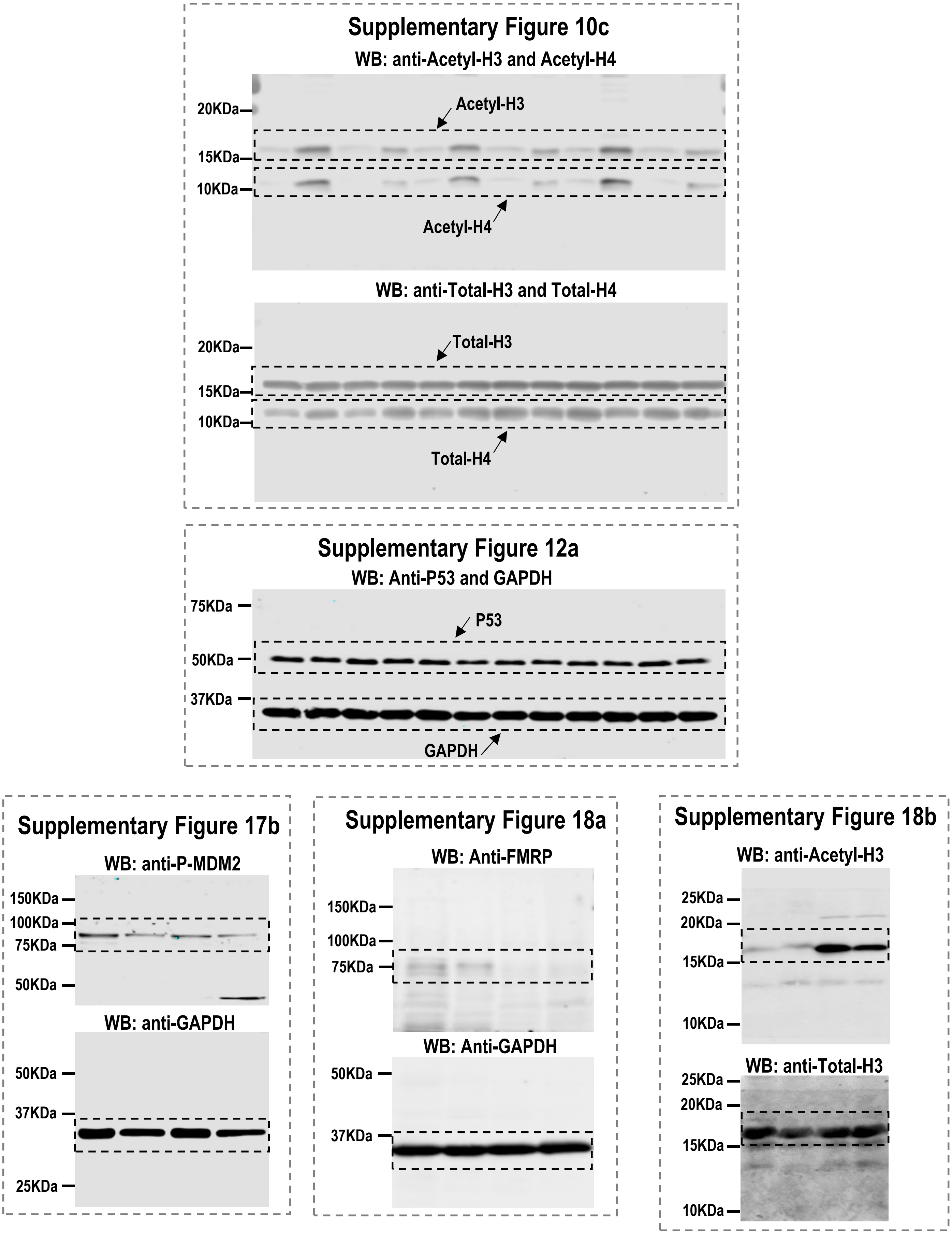

Supplementary Figure 26:The Full blot used for supplementary figures 10b,12a,17b,18a-b

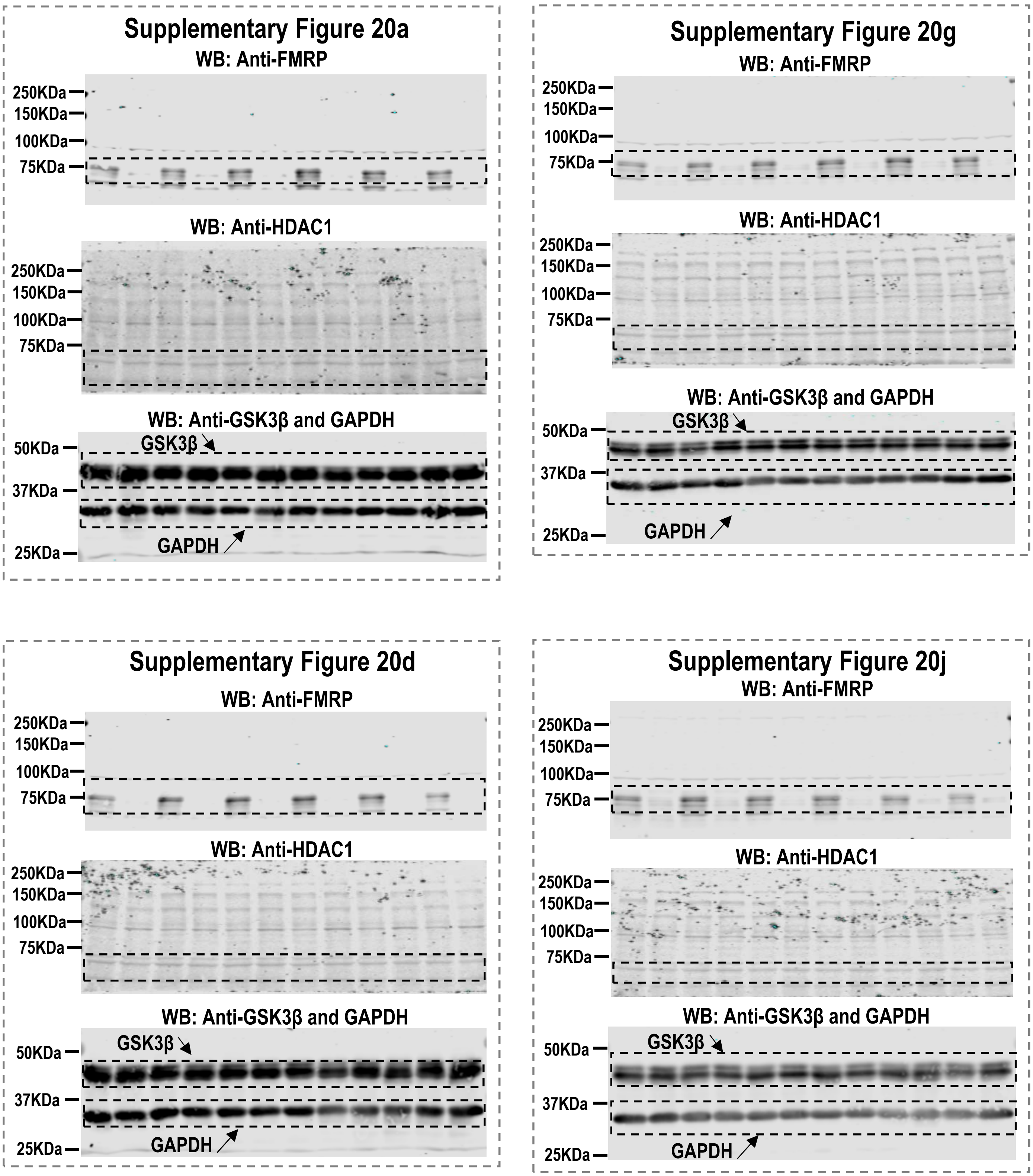

Supplementary Figure 27:The Full blot used for supplementary figure 20
