# Supplementary material for: Reducing histone acetylation rescues cognitive deficits in a mouse model of Fragile X syndrome
Source: Nat Commun. 2018 Jun 27;9:2494. doi: 10.1038/s41467-018-04869-3 (PMC6021376; doi:10.1038/s41467-018-04869-3)
Supplement: Supplementary file 3 — Description of Additional Supplementary Files [file 41467_2018_4869_MOESM3_ESM.pdf]

**Description of Additional Supplementary Files**

File Name: Supplementary Data 1
Description: MDM2 targets or interactors that may regulate adult neural stem cell activation and maintenance.
