# Supplementary material for: Reducing histone acetylation rescues cognitive deficits in a mouse model of Fragile X syndrome
Source: Nat Commun. 2018 Jun 27;9:2494. doi: 10.1038/s41467-018-04869-3 (PMC6021376; doi:10.1038/s41467-018-04869-3)
Supplement: Supplementary file 2 — Peer Review File [file 41467_2018_4869_MOESM2_ESM.doc]

**Full description for Revised Supplementary Figures**

1. In the legend of Supplementary Figure 1, we added “**P<0.01”, based on the Supplementary 1e.
2. In the legend of Supplementary Figure 10, we deleted “*P<0.05”, since there was no present in the figure.
3. In Supplementary figure 13c, we updated the format from “ng/mL” and “ng/g tissue” to “ng mL^-1^” and “ng g^-1^ tissue”, respectively. In the legend of Supplementary Figure 13, we updated the format from “0.4 mg/kg” to “0.4 mg kg^-1^” and from “75 ng/ml” to “75 ng mL^-1^”.
4. In Supplementary figure 14c, we updated the format from “ng/mL” and “ng/g tissue” to “ng mL^-1^” and “ng g^-1^ tissue”, respectively. In the legend of Supplementary Figure 14, we updated the format from “8 mg/kg” to “8 mg kg^-1^” and from “2500 ng/ml” to “2500 ng mL^-1^”.
5. In the legend of Supplementary Figure 18a, we corrected the word from “twp” to “two”.
